# Significantly enhancing human antibody affinity via deep learning and computational biology-guided single-point mutations

**DOI:** 10.1093/bib/bbaf445

**Published:** 2025-09-01

**Authors:** Junxin Li, Chao Zhang, Wei Xia, Hei Wun Kan, Kaifang Huang, Sai Li, Mark Akinola Ige, Qiuliyang Yu, Jiawei Zhao, Xiaochun Wan, John Z H Zhang, Haiping Zhang

**Affiliations:** Center for Protein and Cell-based Drugs, Institute of Biomedicine and Biotechnology, Shenzhen Institutes of Advanced Technology, Chinese Academy of Sciences, 1068 Xueyuan Avenue, Nanshan District, Shenzhen 518055, China; Key Laboratory of Quantitative Synthetic Biology, Shenzhen Institute of Synthetic Biology, Shenzhen Institutes of Advanced Technology, Chinese Academy of Sciences, 1068 Xueyuan Avenue, Nanshan District, Shenzhen 518055, China; Faculty of Synthetic Biology, Shenzhen University of Advanced Technology, 1 Beizhen Road, Xinhu Subdistrict, Guangming District, Shenzhen 518055, China; Faculty of Synthetic Biology, Shenzhen University of Advanced Technology, 1 Beizhen Road, Xinhu Subdistrict, Guangming District, Shenzhen 518055, China; Shanghai Frontiers Science Center of Artificial Intelligence and Deep Learning and NYU-ECNU Center for Computational Chemistry, NYU-Shanghai, 1555 Century Avenue, Pudong New Area, Shanghai 200062, China; Department of Chemistry, New York University, New York, NY 10003, United States; Key Laboratory of Quantitative Synthetic Biology, Shenzhen Institute of Synthetic Biology, Shenzhen Institutes of Advanced Technology, Chinese Academy of Sciences, 1068 Xueyuan Avenue, Nanshan District, Shenzhen 518055, China; Key Laboratory of Quantitative Synthetic Biology, Shenzhen Institute of Synthetic Biology, Shenzhen Institutes of Advanced Technology, Chinese Academy of Sciences, 1068 Xueyuan Avenue, Nanshan District, Shenzhen 518055, China; School of Chemistry and Molecular Engineering, East China Normal University, 138 Tongpu Road, Putuo District, Shanghai 200062, China; Key Laboratory of Quantitative Synthetic Biology, Shenzhen Institute of Synthetic Biology, Shenzhen Institutes of Advanced Technology, Chinese Academy of Sciences, 1068 Xueyuan Avenue, Nanshan District, Shenzhen 518055, China; Faculty of Synthetic Biology, Shenzhen University of Advanced Technology, 1 Beizhen Road, Xinhu Subdistrict, Guangming District, Shenzhen 518055, China; Center for Protein and Cell-based Drugs, Institute of Biomedicine and Biotechnology, Shenzhen Institutes of Advanced Technology, Chinese Academy of Sciences, 1068 Xueyuan Avenue, Nanshan District, Shenzhen 518055, China; University of Chinese Academy of Sciences, No. 1 Yanqihu East Rd, Huairou District, Beijing, China; Sino-European Center of Biomedicine and Health, Institute of Biomedicine and Biotechnology Shenzhen Institute of Advanced Technology, Chinese Academy of Sciences, Shenzhen 518055, China; Faculty of Pharmaceutical Sciences, Shenzhen University of Advanced Technology, 1 Beizhen Road, Xinhu Subdistrict, Guangming District, Shenzhen 518055, China; Center for Protein and Cell-based Drugs, Institute of Biomedicine and Biotechnology, Shenzhen Institutes of Advanced Technology, Chinese Academy of Sciences, 1068 Xueyuan Avenue, Nanshan District, Shenzhen 518055, China; Key Laboratory of Quantitative Synthetic Biology, Shenzhen Institute of Synthetic Biology, Shenzhen Institutes of Advanced Technology, Chinese Academy of Sciences, 1068 Xueyuan Avenue, Nanshan District, Shenzhen 518055, China; Faculty of Synthetic Biology, Shenzhen University of Advanced Technology, 1 Beizhen Road, Xinhu Subdistrict, Guangming District, Shenzhen 518055, China; Shanghai Frontiers Science Center of Artificial Intelligence and Deep Learning and NYU-ECNU Center for Computational Chemistry, NYU-Shanghai, 1555 Century Avenue, Pudong New Area, Shanghai 200062, China; Department of Chemistry, New York University, New York, NY 10003, United States; Faculty of Pharmaceutical Sciences, Shenzhen University of Advanced Technology, 1 Beizhen Road, Xinhu Subdistrict, Guangming District, Shenzhen 518055, China

**Keywords:** single-point mutation, antibody affinity maturation, high-affinity antibody design, evolutionary constraints, microenvironment point mutation, deep learning-based antigen–antibody models

## Abstract

Enhancing antibody affinity is a critical goal in antibody design, as it improves therapeutic efficacy, specificity, and safety while reducing dosage requirements. Traditional methods, such as single-point mutations or combinatorial mutagenesis, are limited by the impracticality of exhaustively exploring the vast mutational space. To address this challenge, we developed a novel computational pipeline that integrates evolutionary constraints, antibody–antigen-specific statistical potentials, molecular dynamics simulations, metadynamics, and a suite of deep learning models to identify affinity-enhancing mutations. Our deep learning framework includes MicroMutate, which predicts microenvironment-specific amino acid mutations, and graph-based models that evaluate postmutation antigen–antibody-binding probabilities. Using this approach, we screened 12 single-point mutant antibodies targeting the hemagglutinin of the H7N9 avian influenza virus, starting from antibodies with initial affinities in the subnanomolar range, with one showing a 4.62-fold improvement. To demonstrate the generalizability of our method, we applied it to engineer an antibody against death receptor 5 with initial affinities in the subnanomolar range, successfully identifying a mutant with a 2.07-fold increase in affinity. Our work underscores the transformative potential of integrating deep learning and computational methods for rapidly and precisely discovering affinity-enhancing mutations while preserving immunogenicity and expression. This approach offers a powerful and universal platform for advancing antibody therapeutics.

## Introduction

**Figure 1:**
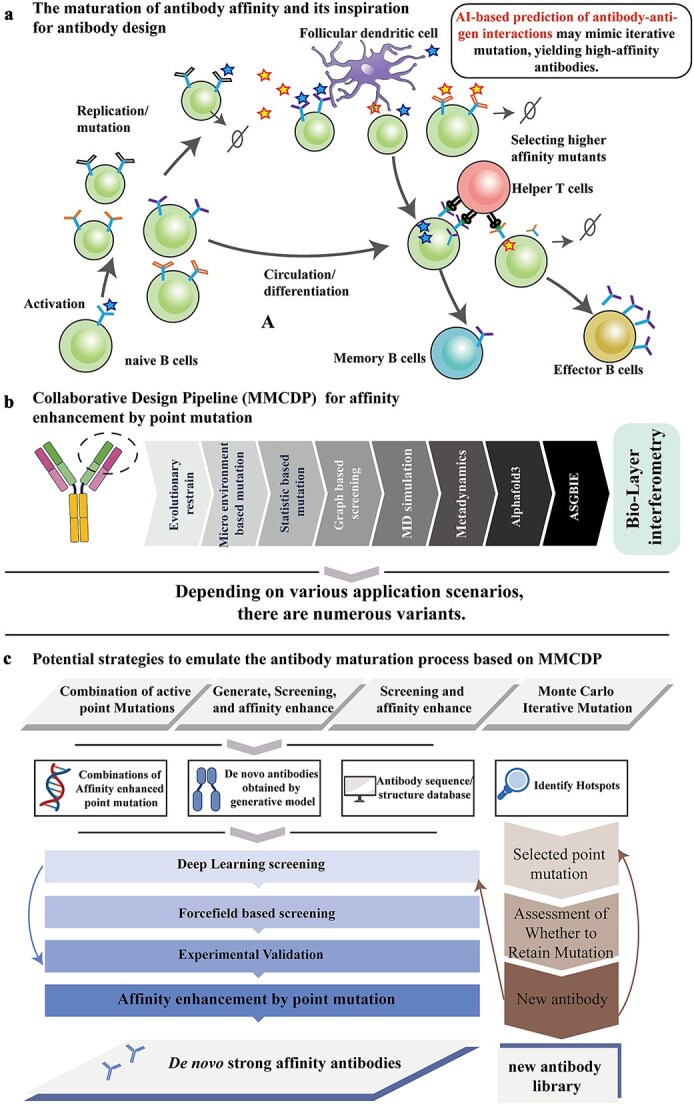
Overview of antibody maturation and the MMCDP. (a) The antibody maturation process. (b) Our proposed MMCDP for affinity enhancement by point mutation. (c) Possible strategies to mimic antibody maturation process based on MMCDP.

The approval rate of biologic drugs, particularly antibodies, has been steadily increasing, highlighting the growing significance of macromolecular therapeutics in modern medicine. In 2023, five antibody-based drugs ranked among the top 10 best-selling drugs worldwide, underscoring their therapeutic and commercial importance. A critical challenge in antibody development, however, is achieving high binding affinity. Many human-derived antibodies exhibit moderate or weak affinities, necessitating further affinity maturation to meet therapeutic standards [1]. Additionally, antibodies obtained from animal models often require humanization, a process that frequently results in a significant loss of binding affinity [2]. Furthermore, the conversion of whole antibodies into smaller fragments, such as Fabs, scFvs, or dsFvs, is often accompanied by a drastic reduction in antigen-binding affinity [3]. These challenges underscore the urgent need for effective affinity maturation strategies to achieve therapeutic-grade binding strengths and optimize the efficacy of antibody-based treatments. A key challenge in antibody engineering is determining whether inactive or low-affinity antibodies can be redesigned to achieve strong activity. During the natural affinity maturation process in humans, antibodies undergo somatic hypermutation, which improves their binding strength through iterative mutations, as illustrated in Fig. 1a. This mechanism offers a valuable framework for designing antibodies *in vitro*. Moreover, the principles underlying affinity maturation in antibodies have significant implications for T-cell receptor engineering, where similar techniques could be applied.

With the rapid growth of antibody sequence data and antibody–antigen interaction (e.g. OAS database [4]) and structural data (e.g. SAbDab [5]), there has been a surge in deep learning-based antibody design studies in recent years. Internationally, Ruffolo *et al.* [6] developed AntiBERTy, a model based on the BERT framework, trained on ~558 million natural antibody sequences from the OAS database using the masked language model approach. In the absence of antibody–antigen complexes, AntiBERTy can identify key hotspot residues in the complementarity-determining region (CDR) regions of antibodies when immune serum data are available. Luo *et al.* [7] proposed DiffAb, a diffusion model-based antibody design tool capable of performing joint sequence-structure design, sequence generation for given scaffolds, and antibody optimization. Barzilay and colleagues developed RefineGNN, comprising two GNNs: one predicts amino acid sequences and the other iteratively refines coordinates based on the sequence [8]. Saka *et al.* [9] employed an LSTM-based generative model for sequence generation and prioritization, effectively discovering antibody sequences with significantly enhanced affinity. Shan and collaborators [10] introduced a geometric deep learning algorithm to enhance antibody affinity for broader and stronger neutralization activities. Gao *et al.* [11] developed a pretrained antibody language model integrated into a specificity antigen–antibody design framework, enabling sequence and structure generation, CDR-H3 design, and antibody optimization.

Despite the rapid development of deep learning-based antibody design methods, most approaches still primarily rely on sequence information. Only a few directly study antibody–antigen interaction interfaces and develop iterative design methods based on these interactions. While AI models can predict general structural changes and functions, they often lack the specificity required for precise affinity enhancement. In addition to emerging deep learning methods, some researchers have successfully applied traditional computational biology approaches to antibody design. For instance, Tharakaraman *et al.* [12, 13] developed a statistical potential-based antibody design method, identifying 10 experimentally validated enhancing mutations from 87-point mutations. Chowdhury and colleagues [14] introduced OptMAVEn-2.0, an antibody design platform that leverages force-field scoring to iteratively optimize antibody properties. This method refines interactions through an energy-based scoring system, providing a robust framework for antibody design and optimization. Desautels *et al.* [15] have used computational methods for restoring the potency of a clinical antibody against Omicron.

In this study, we developed a MicroMutate model and two graph-based antibody–antigen interaction prediction models. These models were integrated with evolutionary constraints, statistical potentials, molecular dynamics simulations, and metadynamics methods to construct an antibody point mutation design pipeline, named the multimethod collaborative design pipeline (MMCDP), as illustrated in Fig. 1b. The evolutionary constraint aids in preventing inexpression issues caused by random mutations, as previously described in our earlier work [16]. This pipeline provides new insights for single-point mutations and serves as a foundation for future multipoint mutations. Inspired by the Roman Testudo formation and the Yuan Yang Formation, military strategies employed by the Roman army and Qi Jiguang’s army in ancient China, respectively. MMCDP leverages the strengths of diverse roles and tactics to achieve superior combat effectiveness. Similarly, MMCDP integrates various self-developed deep learning methods with traditional molecular dynamics simulations and modeling techniques to enable efficient and accurate antibody design. We successfully applied this pipeline to antibody design for two distinct antigens, rapidly identifying enhanced point mutations in both cases. Moreover, such point-based affinity enhancement is at the core of natural antibody maturation (Fig. 1a), suggesting that our methods can play a critical role in strategies designed to mimic the antibody maturation process to obtain fully *de novo* active antibodies, as depicted in Fig. 1c. Effective affinity point mutation plays a significant role in *de novo* antibody design strategies, particularly in integrating active point mutations and employing Monte Carlo-based iterative mutation approaches (Fig. 1c). Additionally, considering the challenges in screening for high-affinity candidates, these mutation strategies can be instrumental in refining the affinity of antibodies obtained through other methods such as *de novo* antibody generation and screening, as well as the screening of large-scale natural antibody libraries (Fig. 1c). Antibody therapeutics hold immense potential across a wide range of diseases, including cancer, viral infections, and autoimmune disorders. These advancements are also expected to benefit modalities such as antibody-drug conjugates, cell therapies, and bispecific antibodies. Our integrated approach, combining multiple computational methods for affinity enhancement, offers a more targeted and efficient strategy for antibody optimization.

## Materials and methods

### Antibody–antigen complex acquisition

The initial structure of hemagglutinin (HA)-antibody complex was obtained from our previous research [16], where the HA was retrieved from the PDB database (PDB ID: 4ln6 [17]). We selected the chain A as the antigen, docked the antibody to the antigen, and carried out a MD simulation to obtain the final frame as the final initial complex for late antigen design. Detailed preparation methods can be found in our previous published work [16].

The initial structure of death receptor 5 (DR5)-antibody complex was obtained from PDB database (PDB ID: 6t3j), we chose chain AB as the antibody (IgG1-hDR5-01) and chain E as the antigen. It should be noticed that we only retained the variable region of IgG1-hDR5-01 during the MD and experimental testing.

### Preparation of sequence library for evolutionary restrain

We have developed an evolutionary restraint-based point mutation method, the detailed protocols of which have been described in our previous paper [16]. The core concept is that by leveraging sequence alignment to extract evolutionary information from the target sequence, mutations are only introduced at positions where a specific amino acid substitution has been historically observed in evolutionary records. This evolutionary constraint minimizes the risk of mutations that could compromise protein expression or stability. However, we used a more comprehensive antibody sequence library as a BLAST search library this time, which was retrieved from https://opig.stats.ox.ac.uk/webapps/ngsdb/paired/, comprising 2 038 528 antibody sequences along with their corresponding CDR regions. Following the removal of redundant entries, distinct datasets for HCDR1, HCDR2, HCDR3, LCDR1, LCDR2, and LCDR3 were compiled, with the following sequence counts: 140 424, 215 636, 1 730 162, 107 215, 4653, and 324 621, respectively. These curated CDR libraries form the basis for our subsequent analytical and design endeavors. Evolutionary conservation data were gathered by performing sequence alignment of homologous antibody by BLASTP [18]. These alignments were used to identify conserved regions and potential mutation hotspots.

### Development and application of microenvironment point mutation model

**Figure 2:**
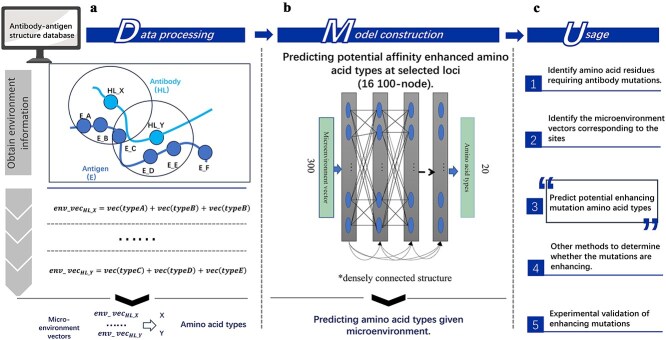
Development of the microenvironment based amino acid mutation model MicroMutate. (a) Input data processing. (b) Model structure and training. (c) Description of possible applications for the MicroMutate model.

We developed the MicroMutate model to predict likely point mutations in the specific microenvironments of antibody–antigen interfaces, as shown in Fig. 2. This model was applied to each candidate complex to identify high-probability affinity-enhancing mutations.

Based on the structural data of antibody–antigen complexes, the microenvironment vectors corresponding to a mutable antibody amino acid were leveraged to identify likely binding amino acid types. There are 5989 complex structures used to generate the mapping pairs of antibody amino acid with its environment amino acids in the interface region, finally obtain 115 369 pairs amino acid–environmental pairs. Deep learning techniques were employed to create pairs between a wide range of antibody amino acids and their corresponding antigen microenvironments. The antigen microenvironment vectors served as training data, while the amino acid types, represented by a one-hot encoding approach, acted as labels for a single-label multiclassification deep learning task.

Label distribution imbalance was addressed by implementing up-sampling techniques to balance the distributions of tags, thereby enhancing model prediction capabilities for potential affinity-enhancing mutations. We employed the RandomOverSampler (random oversampling) from the imblearn library with sampling_strategy = “auto,” which automatically balances classes by oversampling the minority class(es) to match the size of the majority class. This method address label imbalance by randomly duplicating examples from the minority class until the class distribution becomes balanced. We utilize densely connected layers to train the mapping relationship between environment vectors and amino acid types. In practical application, we recommend initially identifying the amino acids to be mutated based on evolutionary information. This integrated approach, combining evolutionary design with complex interface information, holds promise for enhancing the success rate of design efforts.

The performance of MicroMutate over the testing set at different epochs during training is shown in Fig. S1. Given that there are 20 types of amino acids, the random guess accuracy is approximately 0.05. Therefore, an accuracy around 0.1 is significantly better than random guessing. Since each microenvironment may have several preferred amino acids, the labels are not absolutely correct, which explains why the accuracy does not increase further.

### Graph network-based antibody–antigen interaction prediction

**Figure 3:**
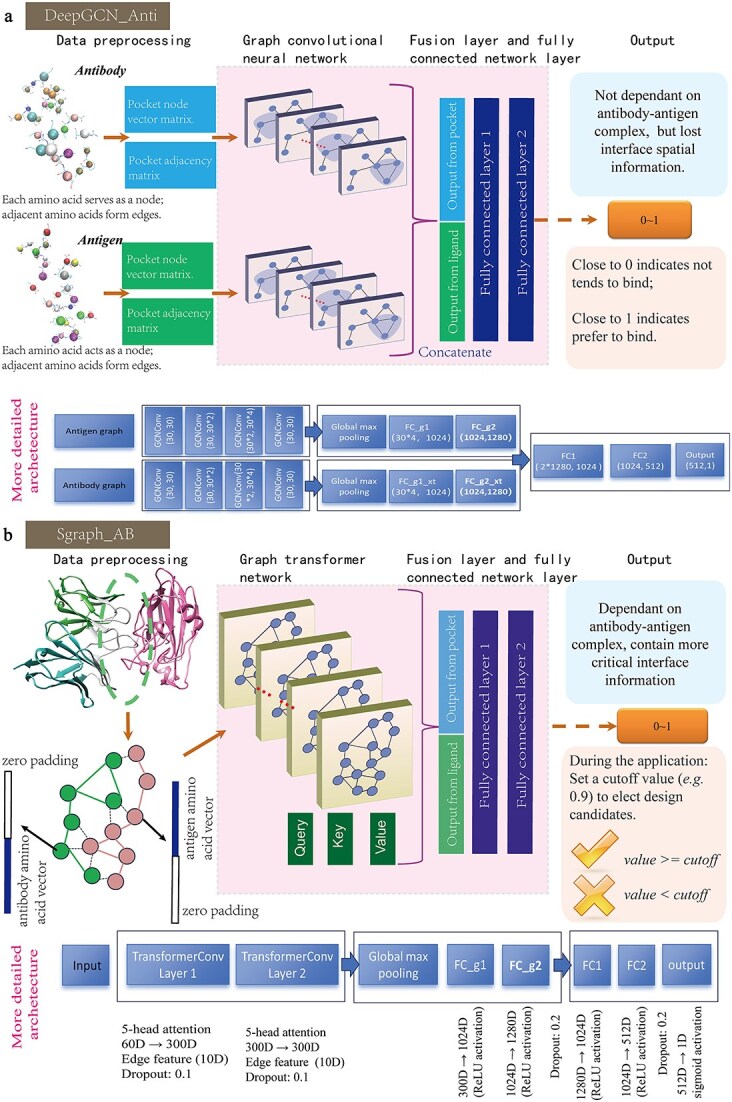
Development of two graph-based antibody–antigen interaction prediction models. (a) Creation of the none-structure dependent DeepGCN_Anti with antibody and antigen binding site graph as input representation. (b) Creation of the interface-dependent model, Sgraph_AB, using a single graph as input representation.

We employed two graph-based models, DeepGCN_Anti and Sgraph_AB, which utilize graph convolutional networks and graph transformer models, respectively, to predict the impact of mutations on antibody–antigen binding. These models interpret the 3D structures of antibody–antigen complexes as graphs, where nodes represent amino acids and edges represent spatial relationships. It is important to note that DeepGCN_Anti has two inputs: one for the antibody side and one for the antigen side, making it independent of complex-specific information. In contrast, Sgraph_AB takes a single graph as input, which contains both the antibody and antigen interface information, and is highly dependent on the antibody–antigen complex. DeepGCN_Anti was developed in our previous work [16], and its model architecture is depicted in Fig. 3a. For more detailed information, please refer to the previous paper [16]. Sgraph_AB, developed in this work, is depicted in Fig. 3b, with additional details provided in the supplemental material section 1. The performance of Sgraph_AB over the testing set at different epochs during training is shown in Fig. S2. According to various performance metrics—such as AUC, true positive rate (TPR) (also named recall), precision, accuracy, and MCC—the model converges around 100 epochs. Therefore, we adopted the trained weights at the 100th epoch as the model’s weights and utilized them in this work. In Table S1, we compare the performance of Sgraph_AB with DeepGCN_Anti. Our Sgraph_AB model trained for 100 epochs demonstrates superior performance across all metrics compared to the final DeepGCN_Anti model trained for 1000 epochs, particularly in TPR.

### Construction of the design pipeline

We integrate various computational methods to construct a design pipeline for identified single-point mutated antibody candidates with potential enhanced affinity. Several combination strategies were use to obtain those candidates.

### Point design of the 3L11 human antibody target HA of H7N9 avian influenza virus

We applied our mutation pipeline to design high-affinity antibodies targeting the 3L11 human antibody. By combining evolutionary restrains, static potential, and deep learning methods (MicroMutate, DeepGCN_Anti, Sgraph_AB), we generated a candidate list. MD simulation and metadynamics were then employed to further refine and obtain final candidates for experimental validation. Detailed procedures are shown at supplementary section 1.

### Point design of DR5 antibody IgG1-hDR5-01 and experimental affinity validation

Similarly, we applied the same pipeline to design and screen antibodies for DR5 protein, mutated from IgG1-hDR5-01.

### Experimental validation

#### Production of antibodies and antigens

Recombinant DR5 was purchased from Sino Biological in China. The genes encoded the IgG1 human antibody and H7N9 HA with a T4 foldon trimerization domain were synthesized and cloned into pcDNA3.1 expression vectors (Invitrogen), respectively. Recombinant proteins were produced by transient transfection of 293F cells sing polyethyleneimine, a nonviral transfection reagent (Sigma-Aldrich), as described previously [19]. Supernatants were collected after 5 days of culture, and HA was purified by nickel affinity chromatography (GE Healthcare).

#### Determination of binding affinity (KD values)

Binding affinities (KD values) were determined by bio-layer interferometry (BLI) using a Gator instrument (PALL ForteBio). Supernatants diluted in sample diluent (SD) buffer [1× PBS, pH 7.4, 1% BSA, and 0.002% Tween 20 (Sigma)] were loaded onto Protein A biosensors and incubated with 500-nM HA or DR5, respectively. All binding data were collected at 30°C. The experimental procedure consisted of four steps:

loading of supernatants onto the biosensor
baseline acquisition for 60 s
association of HA or DR5 to determine the association rate constant (Kon)
dissociation of HA or DR5 to measure the dissociation rate constant (Koff)

The baseline and dissociation steps were carried out in SD buffer alone. The KD value was calculated as the ratio of Koff to Kon.

## Results

### Exploration of affinity-enhanced point mutants of 3L11

**Figure 4:**
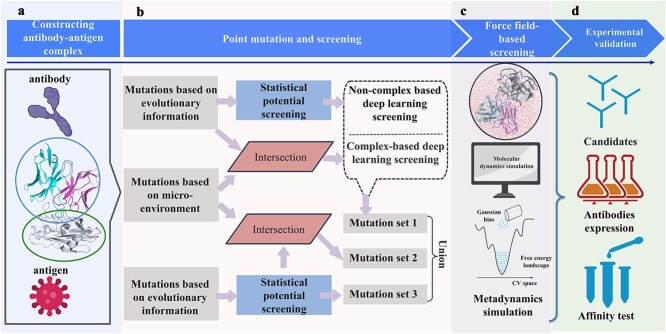
Process of point mutation and screening for 3L11 antibody. (a) Construction of antibody–antigen complex. (b) Point mutation and screening by combining different methods, the first “mutations based on evolutionary information” box represents BLAST CDR analysis, the “mutations based on microenvironment” box corresponds to MicroMutate outputs, the second “mutations based on evolutionary information” box also denotes BLAST CDR outputs, intentionally duplicated for visual representation of combinatorial workflows. (c) Molecular simulation and metadynamics simulation for fine screening. (d) Experimental validation.

Using the design workflow illustrated in Fig. 4, which integrates various computational methods, we identified 12 candidate antibodies with potential enhanced affinity. Among these methods, the statistical potential (developed in our previous work [16]) quantifies pairwise interactions between distinct amino acid types across antigen–antibody interfaces by systematically enumerating proximal residue pairs within interacting regions. Note that in Fig. 4b, both “mutations based on evolutionary information” boxes represent BLAST CDR outputs—duplicated intentionally to visualize distinct combinatorial approaches. Several combination strategies were adopted to obtain these candidates, considering that each method has its own advantages and disadvantages. For candidate selection in Fig. 4b, we implemented method-specific thresholds to filter results from complementary computational approaches, retaining their union as the collective candidate pool. Given the impracticality of fixed cutoffs across diverse systems, we dynamically adjusted these thresholds to ensure downstream experimental validation remained within feasible throughput capacity. To clarify the cutoff selection process in this study, we have incorporated a decision tree plot (Fig. S3; available online at http://bib.oxfordjournals.org/) illustrating the detailed mutation and filtering workflow for the 3L11 antibody. It should be noted that these thresholds are flexible to accommodate computational resource availability. For instance, when limited resources necessitate stricter cutoffs to reduce candidate numbers in later stages, a rigorous threshold may be applied. Conversely, if the user possesses abundant computational resources or a stringent cutoff yields an insufficient number of candidates, we recommend adopting a less strict threshold.

All candidates passed the simulation and metadynamics screening processes, detailed procedures are shown at supplementary section 2 (Fig. S4, available online at http://bib.oxfordjournals.org/; Fig. S5, available online at http://bib.oxfordjournals.org/). Currently the MD simulation only used 20 ns to balance efficiency and accuracy during large-scale screening, but we encourage users to employ longer simulations when computational resources permit. Ultimately, we have obtained 12 antibody candidates for experimental validation. Among these, six mutated antibodies exhibited increased affinity, with four showing significant affinity improvements: L:S93N, L:S93Y, L:G94N, and H:S55N, which displayed affinity enhancements of 2.98-, 2.73-, 2.13-, and 4.62-fold, respectively. Notably, L:G94N was identified based on evolutionary constraints and statistical potentials (Mutation set3 in Fig. 4), while L:S93N originated from evolutionary information and microenvironment point mutations (Mutation set 2 in Fig. 4). L:S93Y and H:S55N were identified through a combination of deep learning and evolutionary data. L:H96N and L:D25S showed only very slight enhancements in affinity. The mutation H:S55N was located on the heavy chain, while L:S93N, L:S93Y, and L:G94N occurred on the light chain.

### Analysing the evolutionary tendency for each position of the six CDRs of the 3L11

**Figure 5:**
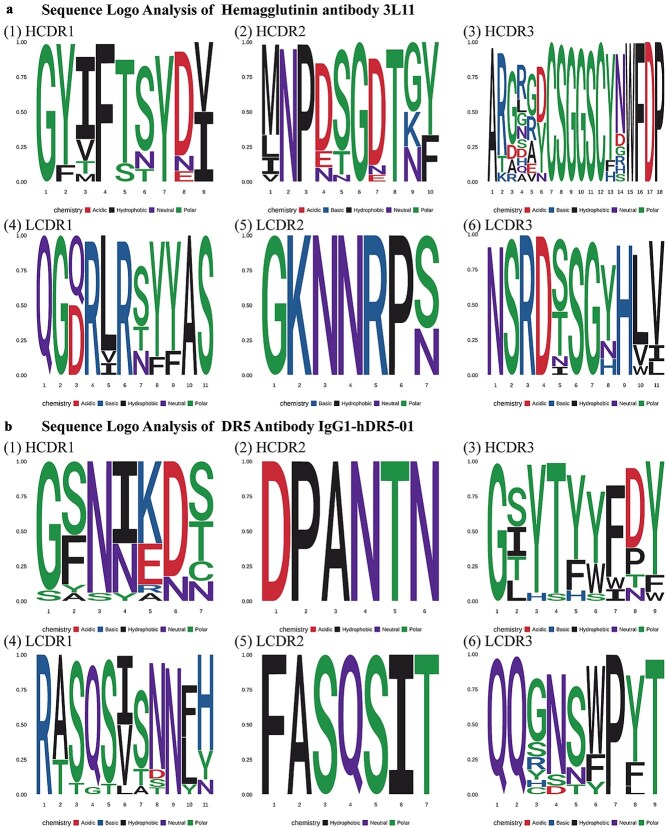
Sequence logo analysis of CDR regions. A percentage cutoff of 0.05 was applied to improve the clarity of the sequence logos. Panel A (1)–(6) depict the amino acid preferences of HA antibody 3L11 for the heavy chain CDR1, CDR2, CDR3 and light chain CDR1, CDR2, CDR3, respectively. Panel B (1)–(6) depict the amino acid preferences of DR5 antibody IgG1-hDR5-01 for the heavy chain CDR1, CDR2, CDR3, and light chain CDR1, CDR2, CDR3, respectively. The sequence logos highlight conserved and variable residues within each CDR, offering critical insights for selecting mutations in the antibody paratope that enhance affinity while maintaining expression levels and structural integrity.

By performing a BLAST search of each CDR of the 3L11 against the corresponding CDR dataset, we obtained the matching sequence pairs. Through these alignment pairs, we determined the amino acid preference, shown as Fig. 5.

### Structural analysis of affinity enhanced mutations in 3L11

**Figure 6:**
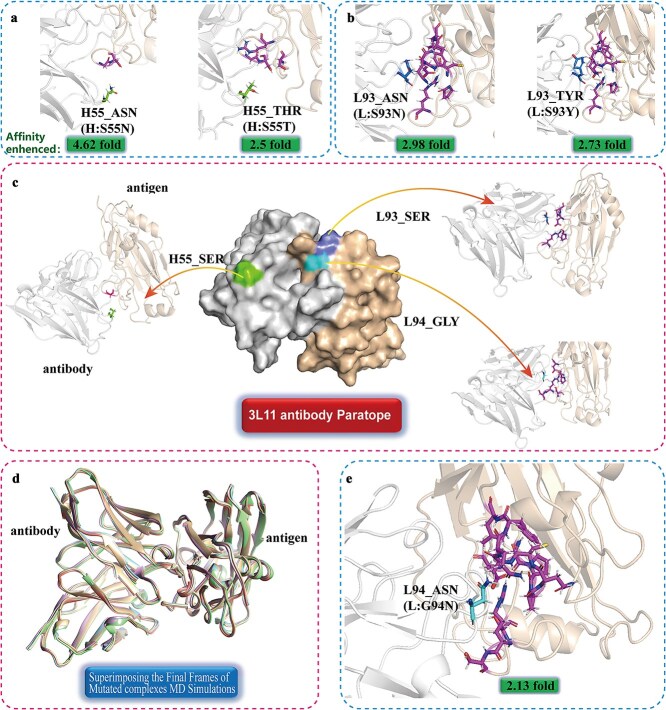
Structure analysis of affinity-enhanced mutations. (a) Affinity-enhanced mutation at position H55 and its neighboring residues on the antigen. (b) Affinity-enhanced mutation at position L93 and its neighboring residues on the antigen. (c) The native 3L11 paratope, highlighting the three positions with enhanced mutations and their original neighboring residues on the antigen. (e) Structure superposition of the final frames from the MD simulations of the affinity-enhances mutated complex. (f) Affinity-enhanced mutation at position L94 and its neighboring residues on the antigen.

To understand the underlying mechanisms of affinity enhancement, we conducted detailed structural analyses of the mutations. The observed affinity improvements could be attributed to factors such as enhanced π–π interactions, altered electrostatic charges, and optimized hydrogen bonding networks at the antibody–antigen interface. For example, mutations H:S55N or H:S55T introduced more antigen residue (with cutoff 0.6 nm), as shown in Fig. 6a and c, leading to increased polar interactions with antigen residues, thus stabilizing the complex. Similarly, mutations in L:S93N or L:S93Y also introduced significantly more residue neighboring (with a cutoff 0.6 nm), as shown in Fig. 6b and c. The L:S93N mutation may introduce more polar interactions, while L:S93Y enhanced π–π stacking interactions and polar interaction, contributing to the substantial affinity gains observed. Similarly, the L:G94N also introduced more neighboring residues, increasing polar or hydrophobic interactions, as depicted in Fig. 6c and f. Additionally, after 20 ns of MD simulation, the mutated antibody–antigen complex showed no significant conformation changes, as shown in Fig. 6e. Interestingly, we observed that the original residues of L93 and H55 were SER, which may be due to the relatively small side chain of SER, allowing for easier enhancement of interactions when replaced with a similar residue with a longer side chain. Moreover, we noted that among the five enhanced mutations, three involved mutations to ASN, indicating that the ASN may often contribute to stronger effects while maintaining stability. ASN’s longer side chain can induce both polar and hydrophobic interactions. The structural insights provide a deeper understanding of why certain mutations led to dramatic affinity improvements, emphasizing the effectiveness of combining computational predictions with evolutionary information to guide antibody design.

### Affinity comparison between human antibodies and their mutants

**Figure 7:**
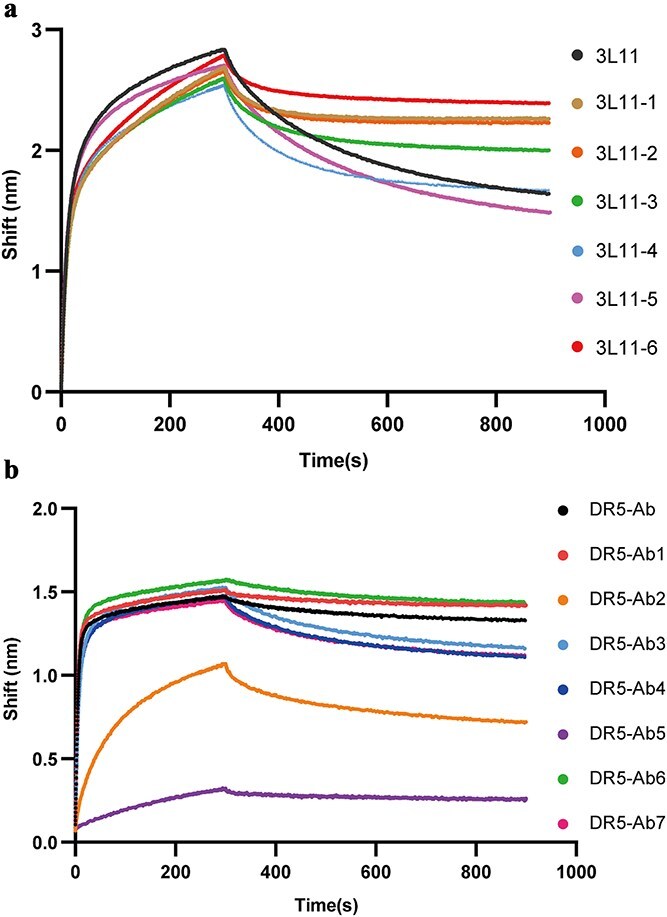
The binding affinities of antibodies determined by BLI. Each antibody in the supernatant was loaded onto protein a biosensor for BLI analysis with 500 nM HA or DR5, respectively. Each curve obtained from the data analysis software consists of an association curve and a dissociation curve. (a) Experimental measurement of the affinity of 3L11 antibody mutates. (b) Experimental measurement of the affinity of DR5 antibody mutates.

The 3L11 antibody, isolated from a patient infected with H7N9 virus, binds to HA with relatively high affinity (KD = 5.32 nM) [20]. However, a 2.5-fold enhancement in affinity was still achieved through a single point mutation (H:S55T) using previously described computational methods [16]. In this study, there were 12 mutants of 3L11 selected through deep learning and computational biology techniques, with their sequences listed in Table S2. To measure and compare the binding affinities between 3L11 and its mutants, BLI was performed using a single concentration of HA antigen and the supernatants from 293F cells transfected with the expression vectors containing antibody genes. As shown in Fig. 7a and Table 1, the affinities of 3L11-1, 3L11-2, 3L11-3, and 3L11-6 increased by 2.98, 2.73, 2.13, and 4.62 folds, respectively, compared to the wild-type 3L11.

**Table 1:** Affinities of mutant antibodies binding to HA.

Antibody	Kon (1/Ms)	Koff (1/s)	KD (M)	Mutation
3L11	1.01E+05	1.38E-03	1.34E-08 (2.95E-09)	Wild type
3L11-1	1.02E+05	5.14E-04	4.52E-09 (2.38E-09)	L:S93N
3L11-2	1.06E+05	5.83E-04	4.95E-09 (2.51E-09)	L:S93Y
3L11-3	1.00E+05	6.89E-04	6.37E-09 (2.53E-09)	L:G94N
3L11-4	1.08E+05	1.26E-03	1.11E-08 (4.19E-09)	L:H96N
3L11-5	1.08E+05	1.41E-03	1.28E-08 (2.46E-09)	L:D25S
3L11-6	1.02E+05	3.37E-04	2.93E-09 (1.61E-09)	H:S55N

Antibodies were loaded onto Protein A biosensors for BLI analysis with 500-nM HA. The measured parameters are listed as follows: Kon is association rate constant; Koff is dissociation rate constant; KD is dissociation constant. The experiment was repeated three times. Data are presented as means or means (SDs).

Encouraged by these results, we applied the same methodology to an anti-DR5 antibody, Chain AB of PDB ID 6t3j [21] to evaluate the generalizability of our approach. Detailed MD and metadynamics to obtain the candidates are shown in Figs S6 and S7. The final seven selected candidate sequences are listed in Table S3. Despite the wild-type DR5 antibody already exhibiting high affinity (KD = 0.3 nM), one mutant (DR5-Ab1) demonstrated a 2.07-fold increase in affinity among the seven point-mutant antibodies selected (Fig. 7b; Table 2). These findings underscore the broad applicability and potential of our method for enhancing antibody affinity across diverse targets.

**Table 2:** Affinities of mutant antibodies binding to DR5.

Antibody	Kon (1/Ms)	Koff (1/s)	KD (M)	Mutation
DR5-Ab	2.65E+05	3.44E-04	1.41E-09 (9.27E-10)	Wild type
DR5-Ab1	2.81E+05	1.74E-04	6.81E-10 (5.05E-10)	H:L100I
DR5-Ab2	4.52E+04	1.69E-03	3.51E-08 (1.46E-08)	H:Y104G
DR5-Ab3	1.86E+05	9.76E-04	5.48E-09 (3.03E-09)	H:I29N
DR5-Ab4	2.12E+05	1.06E-03	5.43E-09 (3.26E-09)	H:I29Y
DR5-Ab5	2.63E+04	5.80E-04	2.14E-08 (9.32E-09)	L:Q91N
DR5-Ab6	2.43E+05	3.18E-04	1.38E-09 (8.99E-10)	L:Q93E
DR5-Ab7	2.49E+05	8.75E-04	3.74E-09 (2.10E-09)	L:Q94Y

Antibodies were loaded onto Protein A biosensors for BLI analysis with 500 nM DR5. The measured parameters are listed as follows: Kon is association rate constant; Koff is dissociation rate constant; KD is dissociation constant. The experiment was repeated three times. Data are presented as means or means (SDs).

## Discussion

Our methodology offers significant value for antibody design by enabling the precise identification of point mutations that enhance antibody affinity. This approach provides a powerful tool for optimizing antibodies for both therapeutic and diagnostic purposes. The ability to systematically improve antibody affinity has the potential to transform the landscape of antibody design, opening new avenues for both academic research and industrial applications.

### Alphafold3’s capabilities and limitations in antibody–antigen interaction prediction

**Figure 8:**
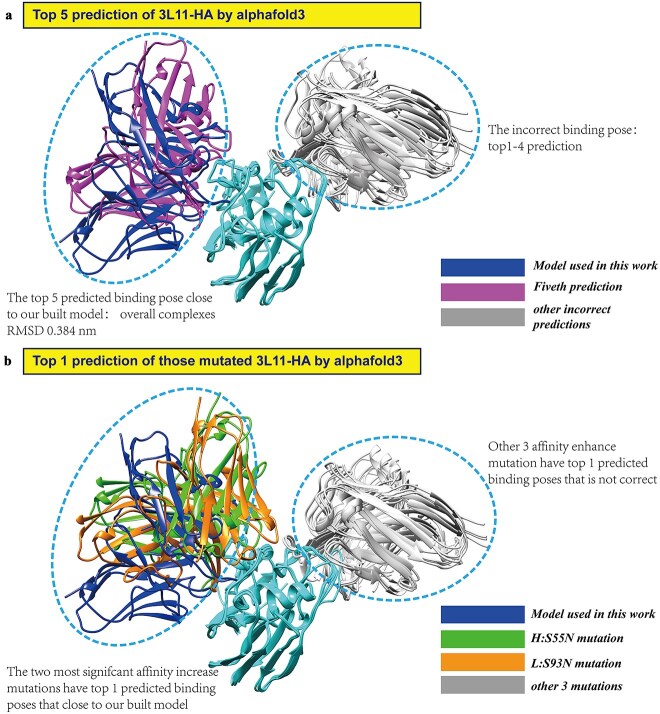
Prediction results of Alphafold3 for 3L11-HA complex and five affinity-enhanced mutated 3L11-HA complexes. (a) The top four predicted conformations of the 3L11-HA complex do not align with the binding site of our reference model. Only the fifth prediction is close to our built model. (b) Two mutated candidates were successfully predicted by Alphafold3 at the top conformation, with binding sites similar to our reference model (blue). Notably, these two mutations (H:S55N and L:S93N) have the largest affinity enhancement.

We employed Alphafold3 [22] (https://alphafoldserver.com/) to predict the 3D structure of 3L11 and its mutated sequences. Among the five predicted structures, of 3L11 only the fifth one was positioned correctly (shown in Fig. 8a), suggesting that Alphafold3 may not be proficient in predicting antibody–antigen complexes. Notably, Alphafold3 accurately identified the correct binding sites for the top two affinity-enhancing mutations, H:S55N and L:S93N (Fig. 8b). This observation implies that Alphafold3 might be particularly adept at recognizing strong affinity binding interfaces. If this is indeed the case, Alphafold3 could prove to be a valuable tool in future research for identifying mutations that strongly bind to their target antigens.

In conclusion, while Alphafold3 showed limitations in predicting the overall structure of antibody–antigen complexes in this particular case, its ability to identify key binding sites for high-affinity mutations suggests potential applications in targeted mutagenesis and structure-based drug design. Future studies should aim to validate these findings and explore the extent of Alphafold3’s capabilities in this domain.

### Exploring the use of computational biology method ASGBIE to elucidate enhanced affinity mutations

While our deep learning and evolutionary-based approaches for identifying affinity-enhanced candidates have proven highly effective, their operation within a “black box” framework limits our understanding of the underlying molecular mechanisms driving these enhancements. To address this limitation, we employed the ASGBIE computational biology method [23–27] to provide mechanistic insights into the affinity improvements observed following five specific mutations. The simulations for both wild-type and mutant forms over 1 μs and analyze the last 100 ns conformations using the ASGBIE method. A residue is considered part of the interface if within a 5Å cutoff. Interaction free energy was decomposed into enthalpy and entropy components. Enthalpy includes van der Waals, electrostatic, and solvent-free energies (both polar and nonpolar), while entropy reflects antibody–antigen interaction entropy. We sequentially scanned all residues on the antibody and antigen interfaces and averaged the results to obtain the interaction free energy between the antibody and antigen for each system. Interestingly, ASGBIE successfully identified four of the five mutations as contributing to reduced binding free energy, mutations H:S55N (−16.81 kcal/mol), H:S55T (−20.39 kcal/mol), L:S93Y (−20.72 kcal/mol), as evidenced in Table 3. However, it failed to accurately predict the L:S93N (−4.10 kcal/mol), which exhibited the second largest increases in binding affinity. Remarkably, we observed a complementary relationship between AlphaFold3 and ASGBIE, where AlphaFold3 accurately predicted the correct binding conformation for L:S93N (as highlighted in Fig. 8), while ASGBIE failed in predicting their affinity change. Conversely, for mutations where AlphaFold3 struggled to predict the overall structure of the antibody–antigen complex, ASGBIE demonstrated correct in predicting the binding free energy changes upon mutation. This suggests that AlphaFold3, a data-driven deep learning model, and ASGBIE, a physics-based computational method, approach the problem of antibody–antigen binding from fundamentally different perspectives, potentially offering complementary strengths.

**Table 3:** The interaction free energy dG between antibody and antigen and all detailed energy terms (kcal/Mol).

Type		dVDW	dEEL	dGB	dNP	dGgas	dGsolv	dH	Interaction entropy	Delta G
WT		−24.20	−37.71	31.33	−2.84	−61.91	28.50	−33.41	18.8	−14.54
Mutation	H:55N	−25.09	−33.00	28.36	−2.44	−58.09	25.93	−32.16	15.36	−16.81
	H:55T	−28.14	−35.15	29.86	−2.64	−63.30	27.22	−36.08	15.69	−20.39
	L:93N	−14.94	−35.84	31.36	−2.37	−50.78	28.99	−21.79	17.68	−4.10
	L:93Y	−29.93	−42.28	35.10	−3.44	−72.21	31.66	−40.56	19.83	−20.72
	L:94N	−32.04	−34.19	26.72	−2.59	−66.23	24.13	−42.10	16.39	−25.71

We also have carried an Alanine Scanning to further analysis the four successful predicted mutates (H:S55N L: H:S55T L:S93Y L:G94N), we assessed each interface residue’s contribution to antibody–antigen binding, shown in Table S4. In the wild-type, H:W50 contributes most significantly, with H:D33, H:W47, H:N52, H:D57, H:D102, H:S104, H:Y109, L:R90, and L:Y95 also key binding sites. Some residues, like L:R28 and L:K50, induce repulsion due to their positive charge. Mutation aims to enhance attraction or reduce repulsion or both. Enhancing attraction can involve strengthening existing positive contributions or creating new attractive sites. For mutation H:S55N, which has a binding similar to wild-type, H:W50’s contribution decreases, but H:Y109’s contribution increases from −5.10 to −6.18 kcal/mol and L:Y30’s from −0.68 to −3.13 kcal/mol. Contributions from L:R90 and L:Y95 also increase, offsetting enhanced repulsion. Notably, both H:S55N and H:S55T mutations diminish contributions from H:W50, H:D102, and H:S104, while enhancing those from L:Y30, L:R90, and L:Y95. Despite both mutations occurring at H:S55, H:Y32’s contribution decreases in H:S55N but increases in H:S55T. Mutations at L:S93 show distinct conformational impacts compared to H:S55 mutations. For instance, H:N35’s contribution increases in H:S55 mutations but decreases in L:S94 mutations, a trend also seen with L:R90. L:S93Y and L:G94N enhance contributions at mutated sites. None of the five mutations significantly affect H:T58, L:N51, or L:N52.

Interactions between amino acids in antibody and antigen were also carried by alanine scanning and double alanine scanning methods. Methods and results are illustrated at Supplementary Section 2, Fig. S8, and Table S5.

This analysis and observation underscore the potential of combining deep learning techniques with computational biology methods to enhance both the predictability of binding affinity and the mechanistic understanding of molecular interactions. By leveraging the strengths of these diverse approaches, future research could enable more precise engineering of antibodies with optimized binding properties, advancing the field of structure-based drug design and antibody therapeutics.

### Potential improvement in MD simulation and metadynamics base screening

The initial 20-ns unbiased MD simulation duration represents a practical balance between computational efficiency and interface assessment for large-scale screening. However, to ensure rigorous validation of the 12 final candidates selected for experimental testing, we extended unbiased MD simulations to 60 ns. These longer simulations demonstrated improved predictive capability: among the four experimentally validated affinity-enhanced mutants, three exhibited exceptionally stable interfaces with consistently low RMSD values (Fig. S9). As quantified in Table S6, the H:S55N_L:G94N and L:S93Y mutants displayed the lowest average RMSD values, correlating with their experimentally confirmed binding improvements. The L:S93N mutant constituted the sole exception, where RMSD fluctuations did not reflect its affinity enhancement.

The metadynamics-based free energy calculations face inherent challenges in achieving full convergence, particularly due to the vast configurational space of antibody–antigen dissociation and the irreversibility of unbinding events. While advanced techniques like funnel metadynamics can improve convergence, their complexity makes them less practical for automated, high-throughput workflows. Our approach prioritizes rapid, semiquantitative estimation of binding tendencies rather than absolute free energy values.

### Advantages of small modification in antibody design

We recognize that small modifications, such as point mutations, to enhance affinity are more likely to preserve an antibody’s immunogenicity, structural stability, solubility, and expression levels. This is particularly important for ensuring the success of antibody drugs in later stages of development. By minimizing significant changes, our approach helps retain the nonaffinity-related properties of the antibody, thereby increasing the likelihood of successful clinical translation.

A particular concern arises from the fact that point mutations introduced during the optimization process may inadvertently reverse humanization efforts, leading to “de-humanized” antibodies. Potential solutions include incorporating evolutionary information specific to human germline sequences during the design process to ensure compatibility with the human immune system.

### Mimic antibody affinity maturation process

Finally, our results provide insights into the antibody affinity maturation process. By simulating point mutations that mimic natural maturation mechanisms, we have demonstrated the possibility of artificially enhancing affinity in a manner that parallels *in vivo* evolution.

A notable advantage of our method is its potential to tailor antibody affinity to specific requirements. Depending on the desired application, our approach allows for the selection of antibodies with varying binding strengths, from low-affinity antibodies suitable for diagnostic applications to high-affinity antibodies optimized for therapeutic purposes. In this study, we investigated two antibodies that already exhibited strong affinity. Despite their initial strong affinity, we were able to enhance their affinity through point mutations, indicating that this approach holds significant potential for optimizing various antibody projects. For antibodies with weaker activity, it is reasonable to expect that this method could achieve even more substantial improvements in affinity.

### Potential improvements

There remains ample room for further refinement and application of this approach. One promising direction is the exploration of combinatorial point mutations, where synergistic effects between multiple mutations could lead to even greater affinity enhancements. Additionally, methods such as pulling simulations and funnel metadynamics offer exciting alternatives for probing the energy landscape of antibody–antigen interactions.

Also, the evolutionary constraints we applied in this work still have limitations. When a CDR sequence can be aligned to many sequences, the current constraints are relatively reasonable. However, when a CDR sequence has few or no matches, the current strategy results in very limited or no mutations at these sites. This may be highly problematic, as the lack of matches could indicate that these regions are less constrained and can tolerate a broader range of mutations. The overly strict constraints led to the identification of only a few mutation sites in this study. For example, in the case of the 3L11 antibody, only two mutation sites were identified, each with just two types of mutations that enhanced affinity by more than 2-fold. This limited the possibility of exploring more combinations and may have hindered the discovery of more potent mutants. A similar issue was observed in the DR5 antibody, where only one site with an enhancing mutation was identified.

To address these limitations, we plan to adjust the constraints based on specific scenarios, allowing for more extensive mutations in regions with few or no matches. Additionally, for CDRs that match few sequences, we could explore the use of generative models to introduce diverse CDR sequences, followed by screening to identify those with enhanced affinity. It is important to note that heavy chain CDRs, particularly HCDR3, which often play a crucial role in antigen binding, may inherently tolerate fewer mutations. If we can identify more sites with enhancing mutations in the future, we can leverage the combination of these mutations to achieve even greater affinity improvements. The success of such combinatorial mutations will likely depend heavily on co-evolutionary relationships to ensure the reliability and stability of the multipoint mutants.

### Possible reasons for the lower success rate of DR5 antibody design compared to 3L11

In this study, we observed that the success rate of DR5 antibody design was lower than that of 3L11, prompting a closer examination of the differences between these two design approaches. The DR5 antibody was designed based on experimentally determined crystal structures, whereas the 3L11 antibody was designed using the final conformation obtained from molecular dynamics simulations of a modeled structure. Although crystal structures are generally considered an ideal starting point for antibody design, our results suggest that even crystal structures may require simulation-based equilibration to better reflect the antibody–antigen conformations in aqueous solution, which would serve as a more optimal design starting point. This is evident from the significant RMSD fluctuations observed in DR5 mutants during simulations (Fig. S6, available online at http://bib.oxfordjournals.org/), compared to the relatively smaller fluctuations in 3L11 (Fig. S4, available online at http://bib.oxfordjournals.org/). Similar findings have been reported in the CASP16 protein–ligand affinity prediction challenge (https://www.predictioncenter.org/casp16/zscores_ligand.cgi, where our Haiping method achieves the Top 1 performance in Stage 1 of protein–ligand affinity prediction), where predictions based on crystal structures did not show improved accuracy (Stage 2). These results suggest that the choice of starting structures in antibody design may need to be re-evaluated, particularly in terms of the potential benefits of simulation-based equilibration.

### Five-fold cross-validation of MicroMutate

To evaluate the robustness of MicroMutate, we implemented five-fold cross-validation while applying random up-sampling to mitigate label imbalance. Performance results (Table S7; Fig. S10) demonstrate outcomes comparable to the original model, achieving a mean test accuracy of 0.099 (random accuracy is 0.05) across all folds at 100 epochs. Model performance remains stable across different folds, with minimal inter-fold variance confirming the reliability of our approach.

### Comparison of cutoff choices for Sgraph_AB

To rigorously assess the appropriateness of the 9Å cutoff for Sgraph_AB, we evaluated three key thresholds: 0.5Å, 0.9Å, and 1.2Å. Performance metrics across epochs are detailed in Table S8 and Figs S11 and S12. Although all cutoffs yield similar overall performance, the 0.5Å model exhibits superior AUC, TPR, accuracy, and MCC, while the 0.9Å and 1.2Å models achieve higher Precision. The 0.9 Å cutoff provides a balanced performance with Precision (0.927), competitive MCC (0.576), and the lowest loss (0.003), justifying its selection as the optimal threshold. We propose that combining predictions from multiple cutoff models could leverage complementary strengths (e.g. 0.5 Å cutoff for sensitivity-driven tasks and 0.9 Å or 1.2 Å cutoff for precision-critical applications.)

### Other potential application of our strategy

The potential applications of our approach extend beyond the optimization of naturally high-affinity antibodies. Antibodies generated by large language models and other generative models, which are becoming increasingly prevalent, often require further affinity maturation. These models can generate antibody sequences or even structures directly tailored to specific antigens, but the affinity of these generated antibodies is not always optimal. Our strategy can be crucial in refining and maturing these antibodies to achieve higher affinity. Similarly, antibodies selected from existing antibody libraries through docking and deep learning techniques can also benefit from point mutation optimization to further enhance their affinity.

The insights gained from microenvironment point mutations in this study may also provide valuable guidance for vaccine protein or enzyme design, where similar principles could be applied to select optimal mutation point and mutation type. This highlights the broader applicability of our approach beyond antibodies, offering a potential new direction for protein engineering.

## Conclusion

This study presents a groundbreaking pipeline, the MMCDP, which integrates evolutionary constraints, statistical potentials, molecular dynamics simulations, metadynamics, and a suite of deep learning models to efficiently and precisely identify affinity-enhancing mutations in antibodies. By leveraging the MicroMutate model and graph-based antibody–antigen interaction prediction models, we have demonstrated the capability to rapidly screen and select mutations that significantly enhance antibody affinity without compromising immunogenicity or expression.

The efficacy of this approach has been validated through successful enhancement of antibody affinity against two distinct antigens: the H7N9 virus and DR5. Notably, the method achieved up to a 4.62-fold increase in affinity for the H7N9 antibody and a 2.07-fold increase for the DR5 antibody, highlighting its broad applicability across different antibody–antigen systems. This underscores the pipeline’s potential to serve as a universal tool for antibody optimization in various therapeutic contexts.

Moreover, given that point mutations are a fundamental aspect of natural antibody maturation, our method provides a computational framework that can effectively mimic and accelerate this process. This capability is particularly valuable in the development of novel antibodies, where rapid identification of optimal mutation sites is crucial.

In comparison to traditional methods, the MMCDP offers a more targeted and efficient strategy for affinity enhancement, reducing the need for extensive experimental screenings and computational resources. This translates into faster drug development cycles and lower costs, making it an attractive option for both academic research and industrial applications.

Looking ahead, the MMCDP lays a solid foundation for more complex antibody engineering tasks, such as multipoint mutation design and the development of bispecific antibodies. The integration of diverse computational techniques within this pipeline not only enhances its predictive power but also opens avenues for continuous improvement and adaptation to new challenges in antibody research.

In summary, this work marks a significant advancement in antibody engineering, offering a powerful and versatile tool for the rapid and precise discovery of affinity-enhancing mutations. The MMCDP has the potential to revolutionize therapeutic antibody development, leading to more effective treatments for a wide range of diseases.

**Key Points**

- The multimethod collaborative design pipeline framework combines microenvironment-aware deep learning (MicroMutate), graph-based binding predictors, molecular dynamics simulations, and evolutionary sequence constraints to systematically identify affinity-enhancing single-point mutations.
- Single-point mutations predicted by the pipeline enhanced binding affinity by 4.6-fold for an H7N9 hemagglutinin antibody (sub-nM to ultra-high affinity) and 2.1-fold for a death receptor 5 therapeutic candidate, demonstrating exceptional precision in refining already optimized biological interfaces.
- Binding free energy decomposition via ASGBIE revealed how mutations redistribute interfacial energy contributions, with successful variants (e.g. H:S55T, L:S93Y) balancing enthalpy–entropy tradeoffs while preserving structural stability.

## Supplementary Material

Supplementary_materials_bbaf445

## Data Availability

The proposed methods, models, and the scripts are available in GitHub public repositories (https://github.com/haiping1010/MMCDP and https://github.com/haiping1010/MD_antibody_antigen/).

## References

[1] Holliger P, Hudson PJ. Engineered antibody fragments and the rise of single domains. *Nat Biotechnol* 2005;23:1126–36. 10.1038/nbt1142. 16151406

[2] Wu H, Nie Y, Huse WD. et al. Humanization of a murine monoclonal antibody by simultaneous optimization of framework and CDR residues. *J Mol Biol* 1999;294:151–62. 10.1006/jmbi.1999.3141. 10556035

[3] Chowdhury PS . Engineering hot spots for affinity enhancement of antibodies. *Methods Mol Biol* 2003, Vol. 207. 10.1385/1-59259-334-8:179. 12412475

[4] Olsen TH, Boyles F, Deane CM. Observed antibody space: a diverse database of cleaned, annotated, and translated unpaired and paired antibody sequences. *Protein Sci* 2022;31:141–6. 10.1002/pro.4205. 34655133 PMC8740823

[5] Schneider C, Raybould MIJ, Deane CM. SAbDab in the age of biotherapeutics: updates including SAbDab-nano, the nanobody structure tracker. *Nucleic Acids Res* 2022;50:D1368–72. 10.1093/nar/gkab1050. 34986602 PMC8728266

[6] Ruffolo JA, Gray JJ, Sulam J. Deciphering Antibody Affinity Maturation with Language Models and Weakly Supervised Learning, arXiv 2021, 10.48550/arXiv.2112.07782.

[7] Luo S, Su Y, Peng X. et al. Antigen-specific antibody design and optimization with diffusion-based generative models for protein structures. *Adv Neural Inf Proces Syst* 2022;35:9754–67.

[8] Jin W, Wohlwend J, Barzilay R. et al. Iterative Refinement Graph Neural Network for Antibody Sequence-Structure Co-Design, arXiv 2022, 10.48550/arXiv.2110.04624.

[9] Saka K, Kakuzaki T, Metsugi S. et al. Antibody design using LSTM based deep generative model from phage display library for affinity maturation. *Reprod Sci* 2021;11. 10.1038/s41598-021-85274-7. PMC7955064 33712669

[10] Shan S, Luo S, Yang Z. et al. Deep learning guided optimization of human antibody against SARS-CoV-2 variants with broad neutralization. Proc Natl Acad Sci USA 2022;119:e2122954119. 10.1073/pnas.2122954119. 35238654 PMC8931377

[11] Gao K, Wu L, Zhu J. et al. Pre-training antibody language models for antigen-specific computational antibody design. KDD, 2023, https://dl.acm.org/doi/10.1145/3580305.3599468.

[12] Tharakaraman K, Robinson LN, Hatas A. et al. Redesign of a cross-reactive antibody to dengue virus with broad-spectrum activity and increased *in vivo* potency. *Proc Natl Acad Sci USA* 2013;110:E1555–64. 10.1073/pnas.1303645110. PMC3637786 23569282

[13] Tharakaraman K, Subramanian V, Viswanathan K. et al. A broadly neutralizing human monoclonal antibody is effective against H7N9. *Proc Natl Acad Sci USA* 2015;112:10890–5. 10.1073/pnas.1502374112. 26283346 PMC4568252

[14] Chowdhury R, Allan MF, Maranas CD. OptMAVEn-2.0: *de novo* design of variable antibody regions against targeted antigen epitopes. *Antibodies* 2018;7;23. 10.3390/antib7030023. PMC6640672 31544875

[15] Desautels TA, Arrildt KT, Zemla AT. et al. Computationally restoring the potency of a clinical antibody against omicron. *Nature* 2024;629:878–85. 38720086 10.1038/s41586-024-07385-1 PMC11111397

[16] Li J, Liao L, Zhang C. et al. Development and experimental validation of computational methods for human antibody affinity enhancement. *Brief Bioinform* 2024;25. 10.1093/bib/bbae488. PMC11446602 39358035

[17] Yang H, Carney PJ, Chang JC. et al. Structural analysis of the hemagglutinin from the recent 2013 H7N9 influenza virus. *J Virol* 2013;87:12433–46. 10.1128/JVI.01854-13. 24027325 PMC3807915

[18] Camacho C, Coulouris G, Avagyan V. et al. BLAST+: architecture and applications. *BMC Bioinform* 2009;10:421. 10.1186/1471-2105-10-421. PMC2803857 20003500

[19] Tom R, Bisson L, Durocher Y. Transfection of HEK293-EBNA1 cells in suspension with linear PEI for production of recombinant proteins. *Cold Spring Harb Protoc* 2008;2008:pdb.prot4977. 10.1101/pdb.prot4977. 21356793

[20] Li J, Yang Y, Wang M. et al. Rapid isolation of a potent human antibody against H7N9 influenza virus from an infected patient. *Antivir Res* 2019;170:104564. 10.1016/j.antiviral.2019.104564. 31336147

[21] Overdijk MB, Strumane K, Beurskens FJ. et al. Dual epitope targeting and enhanced hexamerization by DR5 antibodies as a novel approach to induce potent antitumor activity through DR5 agonism. *Mol Cancer Ther* 2020;19:2126–38. 10.1158/1535-7163.MCT-20-0044. 32847982

[22] Abramson J, Adler J, Dunger J. et al. Accurate structure prediction of biomolecular interactions with AlphaFold 3. *Nature* 2024;630:493–500. 10.1038/s41586-024-07487-w. 38718835 PMC11168924

[23] Kollman PA, Massova I, Reyes C. et al. Calculating structures and free energies of complex molecules: combining molecular mechanics and continuum models. *Acc Chem Res* 2000;33:889–97. 10.1021/ar000033j. 11123888

[24] Massova I, Kollman PA. Computational alanine scanning to probe protein–protein interactions: a novel approach to evaluate binding free energies. *J Am Chem Soc* 1999;121:8133–43. 10.1021/ja990935j.

[25] Duan L, Liu X, Zhang JZH. Interaction entropy: a new paradigm for highly efficient and reliable computation of protein-ligand binding free energy. *J Am Chem Soc* 2016;138:5722–28. 10.1021/jacs.6b02682. 27058988

[26] Yan Y, Yang M, Ji CG. et al. Interaction Entropy for Computational Alanine Scanning. J Chem Inf Model 2017;11:1112–22. 10.1021/acs.jcim.6b00734. 28406301

[27] Liu X, Peng L, Zhang JZH. Accurate and efficient calculation of protein–protein binding free energy-interaction entropy with residue type-specific dielectric constants. *J Chem Inf Model* 2019;59:272–81. 10.1021/acs.jcim.8b00248. 30431271

