# Supplementary material for: Significantly enhancing human antibody affinity via deep learning and computational biology-guided single-point mutations
Source: Brief Bioinform. 2025 Sep 1;26(5):bbaf445. doi: 10.1093/bib/bbaf445 (PMC12400800; doi:10.1093/bib/bbaf445)
Supplement: Supplementary_materials_bbaf445 [file supplementary_materials_bbaf445.docx]

**Supplementary material section 1:**

**Detailed Methods for SGraph_AB**

***Data Preparation***

The model was trained to distinguish between two types of data: actual structure of antibody-antigen pairings, which were treated as positive data, and zdock-based cross-docking of unrelated antibody-antigen complexes within defined epitope-paratope interfaces, which were treated as negative data. We prepared data for training and testing, with 5,000×5 positive data and 22,000 negatives for training, and 895×5 positive data and 3,348 negative data for testing. It should notice that we replicated the positive training and tested data 5 times in the training and testing for the data balance between positive and negative data. We implemented five-fold replication of negative data to mitigate class imbalance, reflecting the biological reality where non-binding antibody-antigen pairs vastly outnumber functional binders - mirroring the inherent challenge of identifying functional antibodies in random screening. While current evidence supports this five-fold replication strategy, we acknowledge the empirical nature of this parameter choice. Future studies should systematically evaluate replication ratios against experimental datasets to optimize generalizability.

***Input Data Representation***

We represented interface amino acids of antibody and antigen as nodes in our graph. Edges were formed based on adjacency: an edge was defined between amino acids Cα atoms of antigen and amino acids of antibody if the distance was less than 9 Å. It is important to choose a proper cutoff, although based on our experience in previous protein-ligand interaction, 9 Å can cover most important interaction and keep a good balance in accuracy and efficiency, but we not sure whether this is the best choose, so in future we can also test with 5 or 12 Å. In this approach, the nodes representing amino acids in the antibody were represented using 30-dimensional word vectors retrained with mol2vec, concatenated with an additional 30-dimensional vector of zeros. Conversely, the nodes representing amino acids in the antigen were represented using a 30-dimensional vector of zeros concatenated with the 30-dimensional word vectors retrained with mol2vec. This method allows the model to effectively distinguish between amino acids from the antibody and those from the antigen. The edge attributes were represented using the one-hot encoding of the Cα-Cα distance (we converted the distance to an integer and then mapped it to a one-hot encoding.). To integrate amino acid and small molecule nodes into a unified graph, we standardized their vector lengths to 75 dimensions: amino acid vectors were padded from the back and small molecule vectors were padded with zeros from the front.

***Model architecture***

We use a graph to represent the interface amino acids and small molecule atoms as nodes, with their adjacency relationships serving as edges. The model is built using PyTorch and PyTorch Geometric, incorporating a sophisticated architecture to capture complex interactions between nodes.

The architecture includes two Transformer Convolution layers (TransformerConv). The first Transformer Convolution layer has 5 attention heads, with both the input and output dimensions set to num_features_xd (60). The second Transformer Convolution layer also has 5 attention heads, but with an input dimension of 5 * num_features_xd (60) and an output dimension of num_features_xd (60). Each Transformer Convolution layer includes a dropout rate of 0.1 to prevent overfitting and accounts for edge dimensions (10). After the transformer layers, the model processes the graph data through a series of linear layers. The first linear layer has an input dimension of 5 * num_features_xd (60) and an output dimension of 1024, followed by a ReLU activation function. The second linear layer has an input dimension of 1024 and an output dimension of 1024, followed by a dropout operation with a rate of 0.2. The third linear layer maintains both input and output dimensions of 1024. The fourth linear layer has an input dimension of 1024 and an output dimension of 512. Finally, the fifth linear layer has an input dimension of 512 and an output dimension of 1, with a sigmoid activation function to ensure the output is in the range of 0 to 1.

**Supplementary material section 2:**

**Detailed procedure of molecular dynamics and metadynamics simulation**

We first carried molecular dynamics simulations for those design antibody-antigen complexes (to facilitate the process, we used 20ns simulation time). We selected those antibody-antigen pairs that have relatively low RMSD during MD simulation for further binding free energy evaluation. Binding free energy calculation can be estimated by metadynamics simulations to explore whether antibody-antigen will bind in solution. Metadynamics relies on addition of a bias potential to sample the free energy landscape along a specific collective variable of interest [1]^,^[2]. Note that the binding free energy calculations from Metadynamics may only be suitable for detect the general trend of binding in virtual screening. For the original 3L11-antigen complex was from the zdock, and its last frame of 40 ns simulation was used as starting conformation of mutations. The mutation conformation was refined by pdbfixer tool and used for late-stage MD simulation. It should notice that the amino acid that far away from the binding interface of the antigen (residue ID 1 to 45 and 259 to 316) was deleted to facilitated the MD simulation. For Dr5-antibody structure, we obtain its antibody-antigen complex directly from AB and E chains of PDB 6T3J, and it was used as starting conformation of mutations.

MD simulation was carried out by Gromacs with AMBER-99SB force field [3,4]. Firstly, we created a dodecahedron box and put the antibody-antigen complex at the center. A minimum distance from the protein to box edge was set to 1 nm. We filled the dodecahedron box with TIP3P water molecules [5], the counter ions were added to neutralize the total charge using the Gromacs program tool [6]. The long-range electrostatic interactions under the periodic boundary conditions was calculated with Particle Mesh Ewald approach [7]. A cutoff of 14 Å was used for van der Waals non-bonded interactions. Covalent bonds involving hydrogen atoms were constrained by applying the LINCS algorithm [8].

We performed the energy minimization steps with a step-size of 0.0001ns, 40 ps simulation with isothermal-isovolumetric ensemble (NVT), and 10ns simulation with isothermal-isobaric ensemble (NPT) for water equilibrium. After that, a 20ns NPT production run (step size 2 fs) was carried out. The Parrinello-Rahman barostat and the modified Berendsen thermostat were used for simulation with a fixed temperature of 308 K and a pressure of 1 atm. RMSD and hydrogen bond number of the trajectory were calculated using Gromacs tools.

The simulation was continued using the metadynamics approach for exploring the free energy landscape. The interface coordination number of atoms of antigen-antibody complex was used as collective variable (CV). The antigen-antibody interface coordination numbers correlate with the numbers of atom contact, and larger coordination number usually indicates that antigen-antibody is in binding state.

The coordination number *C* is defined as follows by Plumed:

$C=\sum_{i\in A}\sum_{j\in B}S_{ij}$ (1) and

$S_{ij}=\frac{1-\left(\frac{r_{ij}-d_{0}}{r_{0}}\right)^{n}}{1-\left(\frac{r_{ij}-d_{0}}{r_{0}}\right)^{m}}$ (2)

In the simulation, *n* was 8, *m* was 12, $d_{0}$ was 0 nm and $r_{0}$ was 0.5 nm. $d_{0}$ is a parameter of the switching function. $r_{ij}$ is the distance between atom *i* and atom *j*. The degrees of contacts between two groups of atoms can be estimated by above function(1) [9]. Metadynamics simulation for each antibody-antigen system was performed for 20 ns. During the metadynamics simulation, Gaussian values were deposited every 1 ps with a height of 0.2 kJ/mol. The widths of the Gaussians were 3 for the coordination number. The free energy landscapes of the metadynamics simulations along the CV were generated by the Plumed program and plotted using Gnuplot [10].

**Supplementary material section 2:**

**Interactions between amino acids in antibody and antigen**

If calculating the multi-molecule enzyme system, the alanine scanning method can also be applied to calculate the binding energy between molecules. The binding energy is important for determining the interactions between multi-molecules and improving the efficiency of complex functional execution in multi-enzyme systems. The binding free energy between molecules is obtained by the following formula:

$\begin{matrix} \Delta\Delta G_{bind}^{x\to a}=\Delta G_{bind}^{x}-\Delta G_{bind}^{a} \\ \Delta G_{bind}^{x}=\Delta G(A(x)-B(y))=\Delta G_{gas}(A(x)-B(y))+\Delta G_{sol}(A(x)-B(y)) \\ \Delta G_{bind}^{a}=\Delta G(A(a)-B(y))=\Delta G_{gas}(A(a)-B(y))+\Delta G_{sol}(A(a)-B(y)) \end{matrix}$ (1)

Before the Double Alanine-Scanning, the y has been mutated to a (Alanine), and the binding free energy is given by

 (2)

The interaction between x and y is expressed by

 (3)

Based on this, we employed the Double Alanine Scanning method (Fig. S7 and Eq.1-3) to analyze interactions between all amino acid pairs at the antibody-antigen interface for both wild-type and mutant forms. This approach enabled us to assess how each mutation affects interactions between specific amino acid pairs at the interface. Such detailed analysis provides insights into the microscopic mechanisms by which mutations influence binding.

In the wild-type, the GH:D33-A:S152 pair contributes most significantly to binding, with an energy of -1.54 kcal/mol. This pair forms four hydrogen bonds, two between the backbone and side chain, and two between side chains, with an average occupancy of 65.5%, an average distance of 3.06 Å, and a bond angle of 156.80°. The GH:W50-A:R148 pair is the next most significant, contributing -1.12 kcal/mol, with one hydrogen bond at 42% occupancy. The charged group of R interacts with the centroid of W's side chain ring at a distance of 5.59 Å, creating a notable cation-π interaction. The GH:D102-A:K159 pair contributes -1.06 kcal/mol, with favorable electrostatic interactions, but the charged centers are 5.06 Å apart, resulting in only two side-chain hydrogen bonds with 33% occupancy. Additionally, alkane-π interactions (L:Y31-A:R265 at -0.73 kcal/mol and L:Y95-A:R148 at -0.64 kcal/mol) significantly support antibody-antigen binding. Repulsion between residues of the same charge hinders binding, as seen with pairs like GH:D102-A:D264 and GH:R26-A:R149, contributing 0.53 and 0.36 kcal/mol, respectively. Beyond electrostatic repulsion, close proximity of residues can increase van der Waals repulsion, further disrupting binding. This is exemplified by GH:S104-A:D264, contributing 0.41 kcal/mol, despite forming two hydrogen bonds.

The H:S55N mutation increases the side chain volume at residue 55, impacting the interface. However, since most interface residues are located in flexible loop regions, a mutation at one site can influence the conformation of other interface residues, affecting antibody-antigen binding. Thus, we performed Double Alanine Scanning calculations on systems with mutations like H:S55N. Based on interaction free energies from the mutated trajectories, we compared the relative positioning of antibody interface residues with antigen residues and observed changes in interaction free energy. Specifically, a negative dDist value indicates that the mutation decreases the distance between residues R1-R2, while a positive value indicates an increase. A negative ddH value suggests enhanced binding between the residues due to the mutation. We found that mutations at H:S55 significantly increased the cation-π interaction between H:W50-A:R148, as indicated by negative dDist values, showing their closer proximity. Additionally, the alkane-π interaction between L:Y30-A:Q86 was strengthened due to reduced distance, promoting antibody-antigen binding. When L:S93 is mutated to TYR, the increased volume allows direct antigen interaction, strengthening the L:Y93-A:Q86 alkane-π interaction by 1.28 kcal/mol. The L:G94N mutation notably enhanced the H:W50-A:R148 cation-π interaction by 2.93 kcal/mol. It suggests that increasing residues with aromatic sidechain at the interface or altering residues to promote π-alkane or π-cation interactions with antigen residues can effectively enhance binding.

**Reference**

1. Laio A, Gervasio FL. Metadynamics: a method to simulate rare events and reconstruct the free energy in biophysics, chemistry and material science. Reports Prog. Phys. 2008; 71:126601

2. Saleh N, Ibrahim P, Saladino G, et al. An Efficient Metadynamics-Based Protocol To Model the Binding Affinity and the Transition State Ensemble of G-Protein-Coupled Receptor Ligands. J. Chem. Inf. Model. 2017; 57:1210–1217

3. Hess B, Kutzner C, Spoel D Van Der. GROMACS 4: algorithms for highly efficient, load-balanced, and scalable molecular simulation. J. Chem. 2008;

4. Hornak V, Simmerling C. Generation of accurate protein loop conformations through low-barrier molecular dynamics. Proteins Struct. Funct. Genet. 2003;

5. Jorgensen WL, Chandrasekhar J, Madura JD, et al. Comparison of simple potential functions for simulating liquid water. J. Chem. Phys. 1983; 79:926–935

6. Van Der Spoel D, Lindahl E, Hess B, et al. GROMACS: Fast, flexible, and free. J. Comput. Chem. 2005;

7. Darden T, York D, Pedersen L. Particle mesh Ewald: An N ⋅log( N ) method for Ewald sums in large systems. J. Chem. Phys. 1993; 98:10089–10092

8. Hess B, Bekker H, Berendsen HJC, et al. LINCS: A linear constraint solver for molecular simulations. J. Comput. Chem. 1997; 18:1463–1472

9. Tribello GA, Bonomi M, Branduardi D, et al. PLUMED 2: New feathers for an old bird. Comput. Phys. Commun. 2014;

10. Williams T, Kelley C, Campbell J, et al. Gnuplot 4.6. Softw. Man. 2012;

**Supplementary Figures:**

**
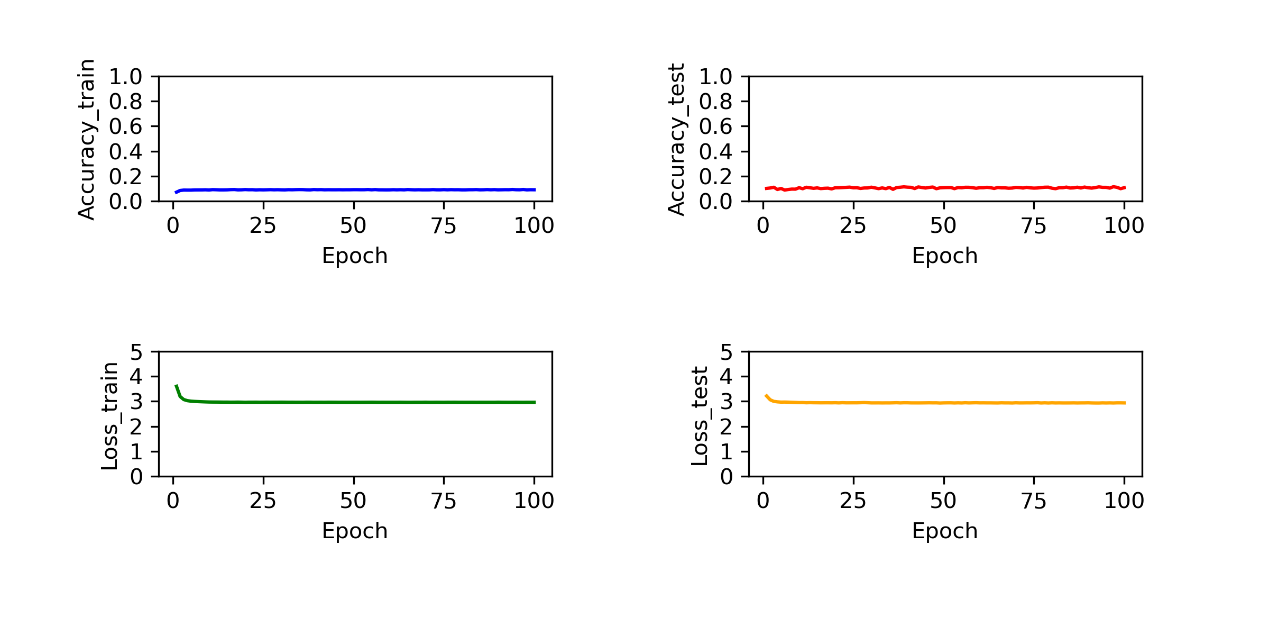
**

**Fig. S1. the accuracy and loss of MicroMutate over the training and testing set at different epochs during the training. Since there are 20 type of amino acid, random guess accuracy is around 0.05, so the accuracy around 0.1 much better than random guess.**

**
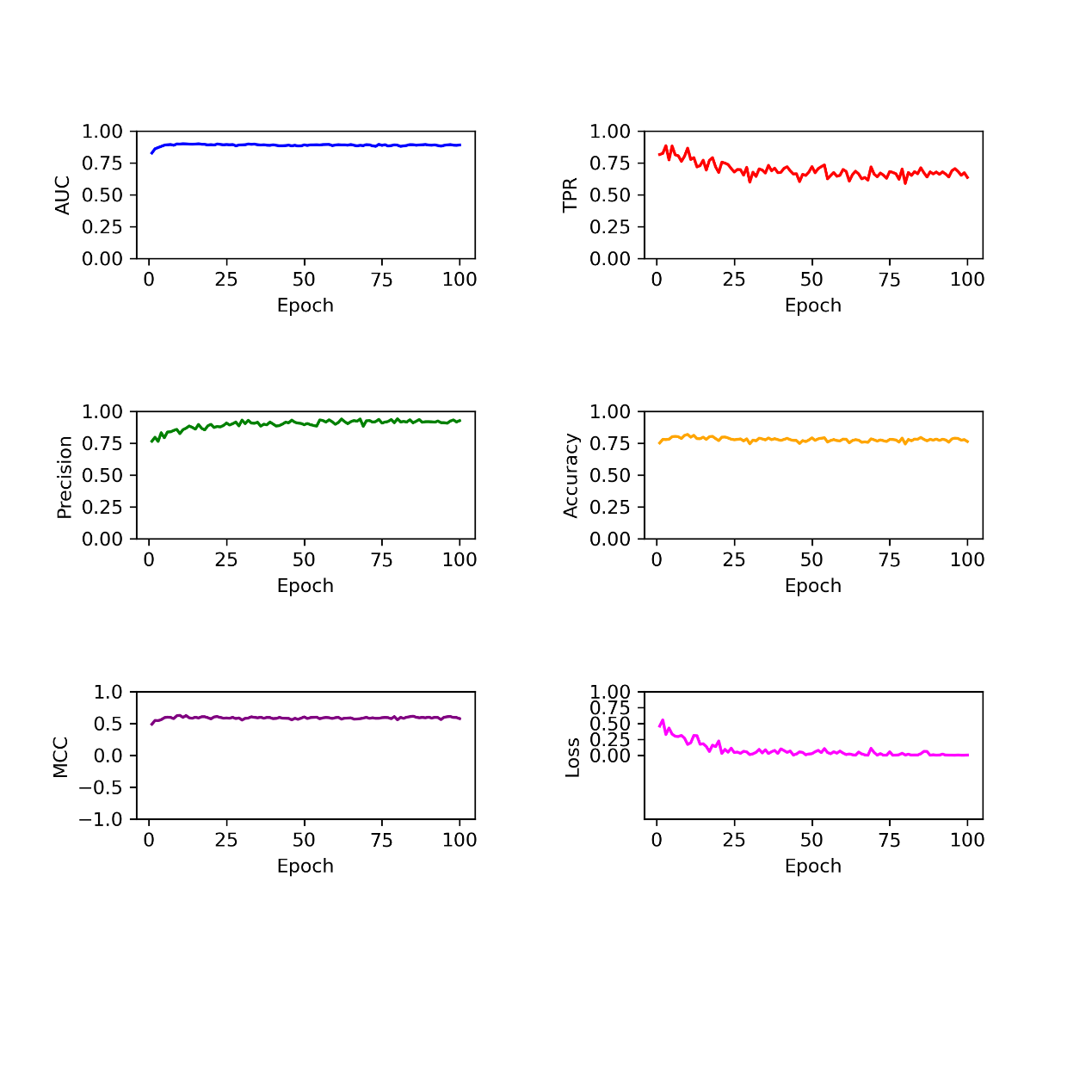
**

**Fig. S2. the performance of Sgraph_AB over the testing set at different epochs during the training.**

**
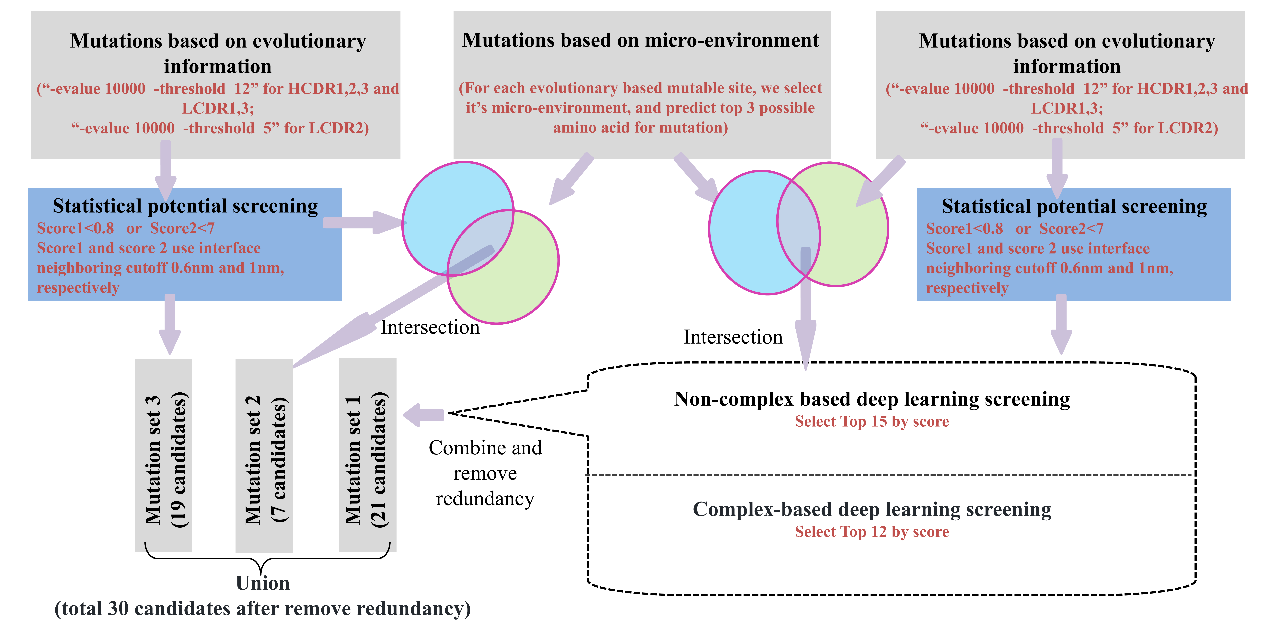
**

**Fig. S3. The detailed mutating and filtering process for 3L11 antibody.**

**
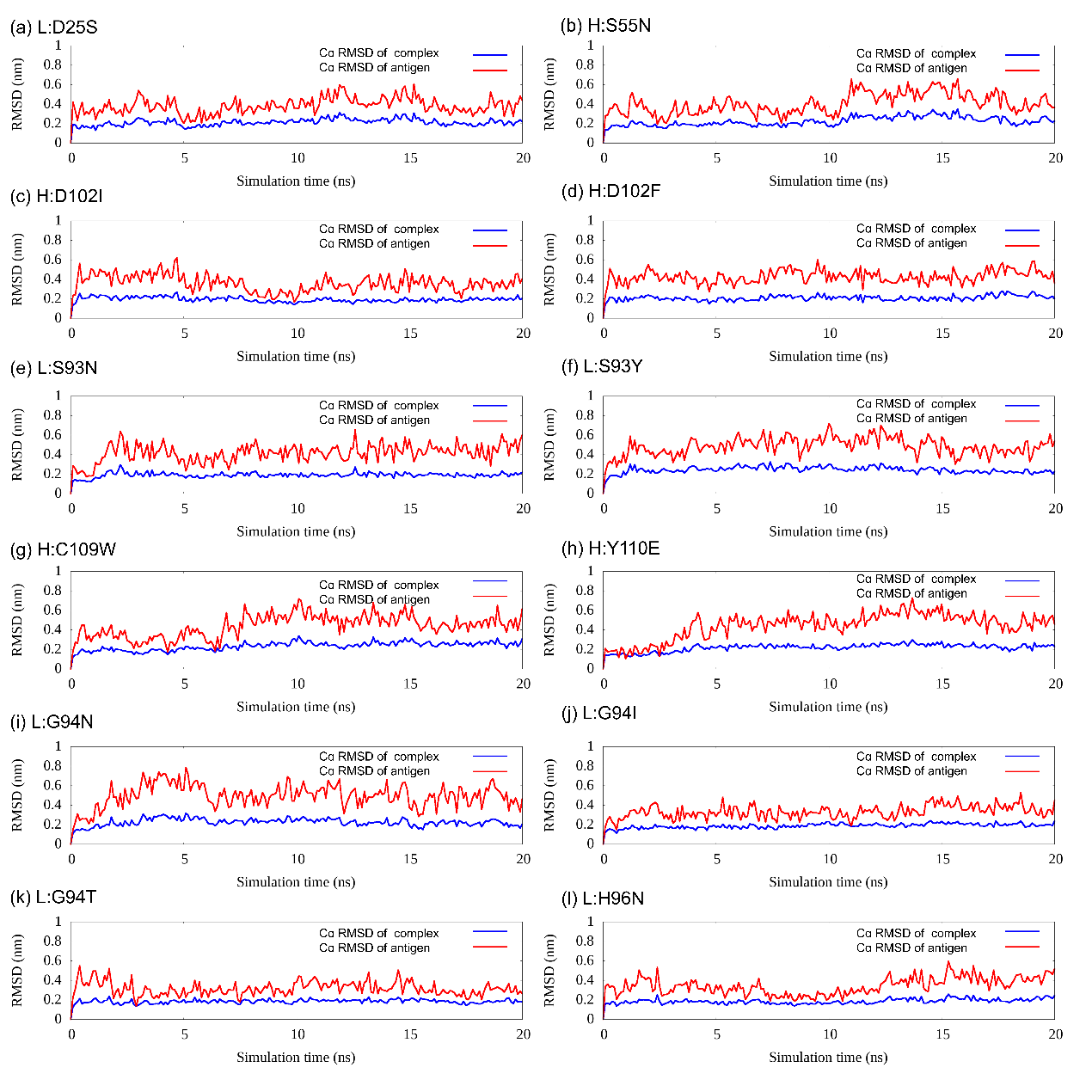
****Fig. S4. The RMSD value of antigen by fitting antibody** **and RMSD of complex during MD simulation for the 3L11 designs.**

**
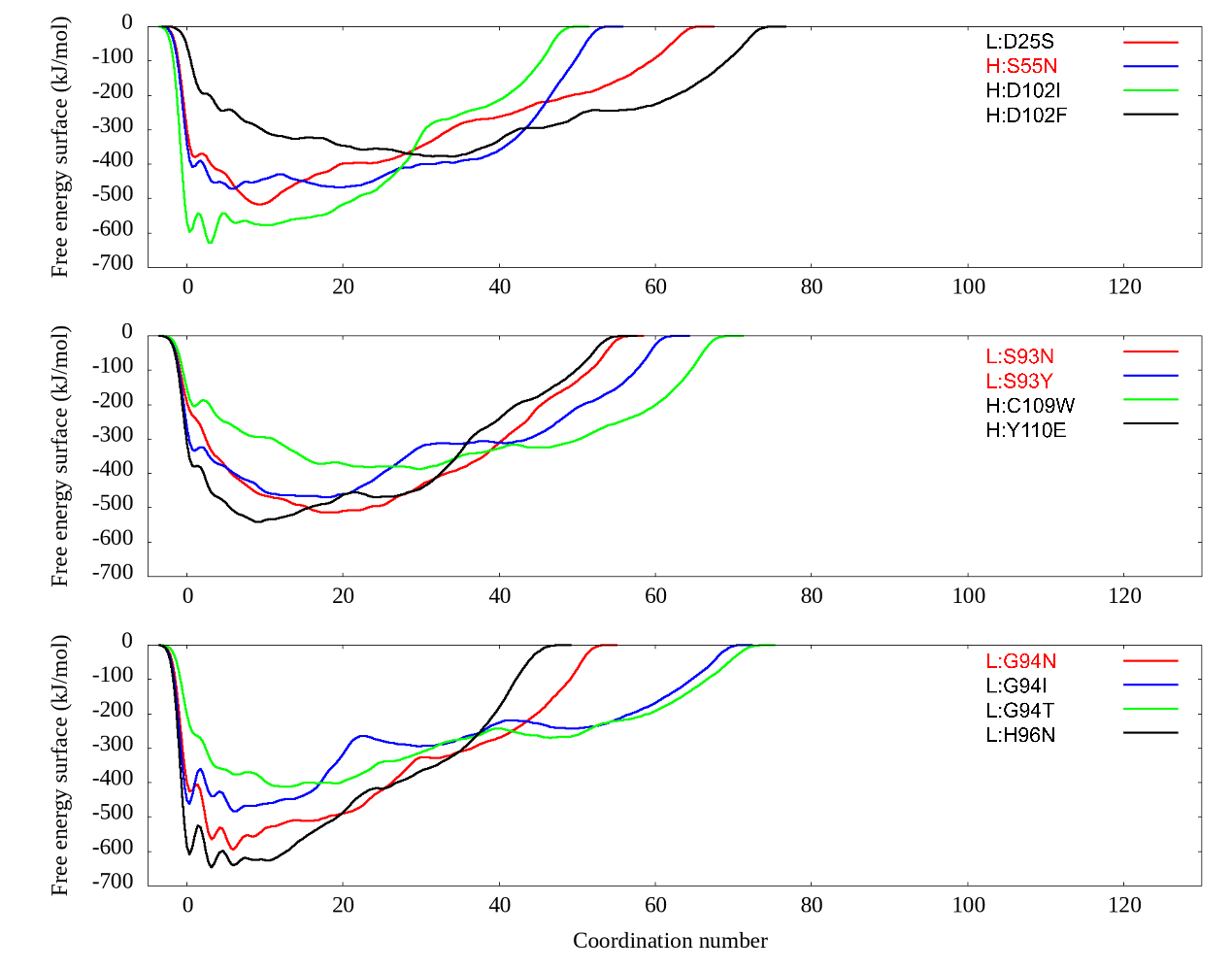
**

**Fig. S5. The free energy profiles caculated by metadynamics for the 3L11 design.**

**
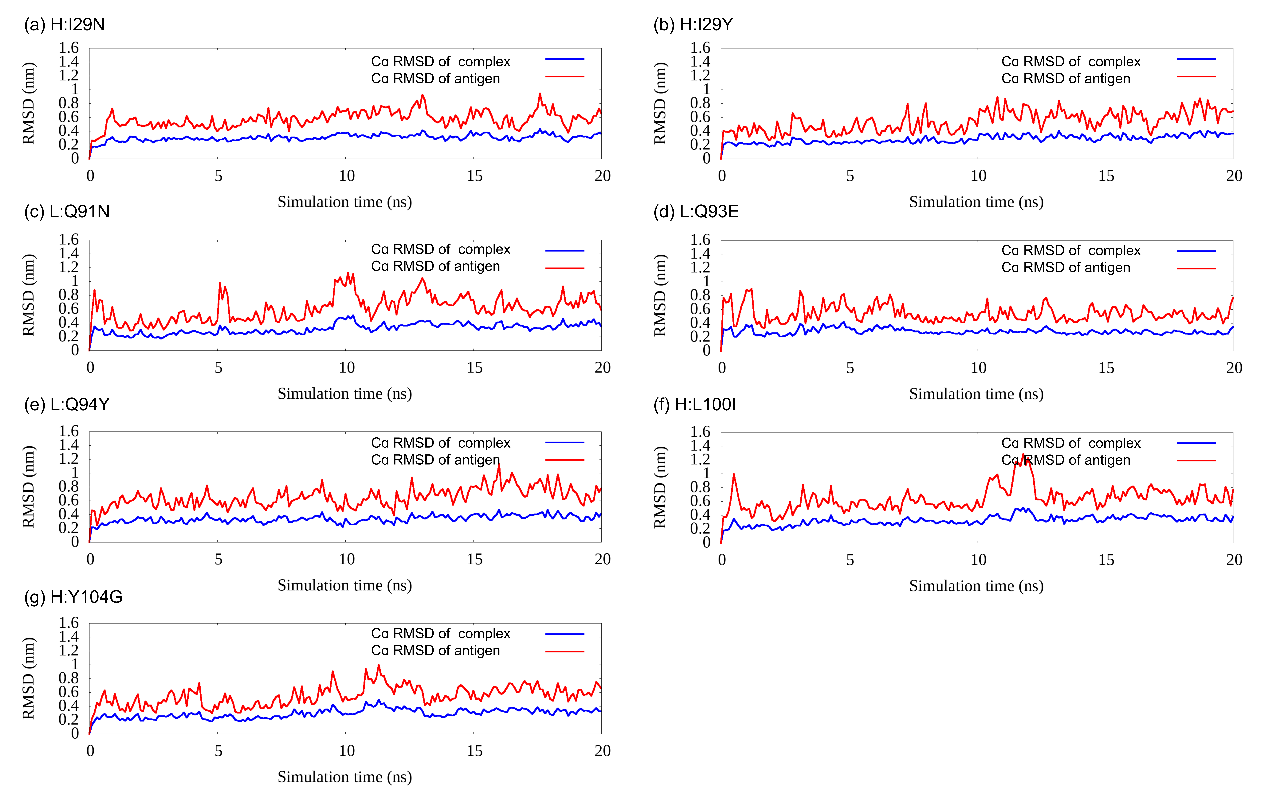
**

**Fig. S6. The RMSD value of antigen by fitting antibody and RMSD of complex during MD simulation for the mutated Dr5 antibodies.**

**
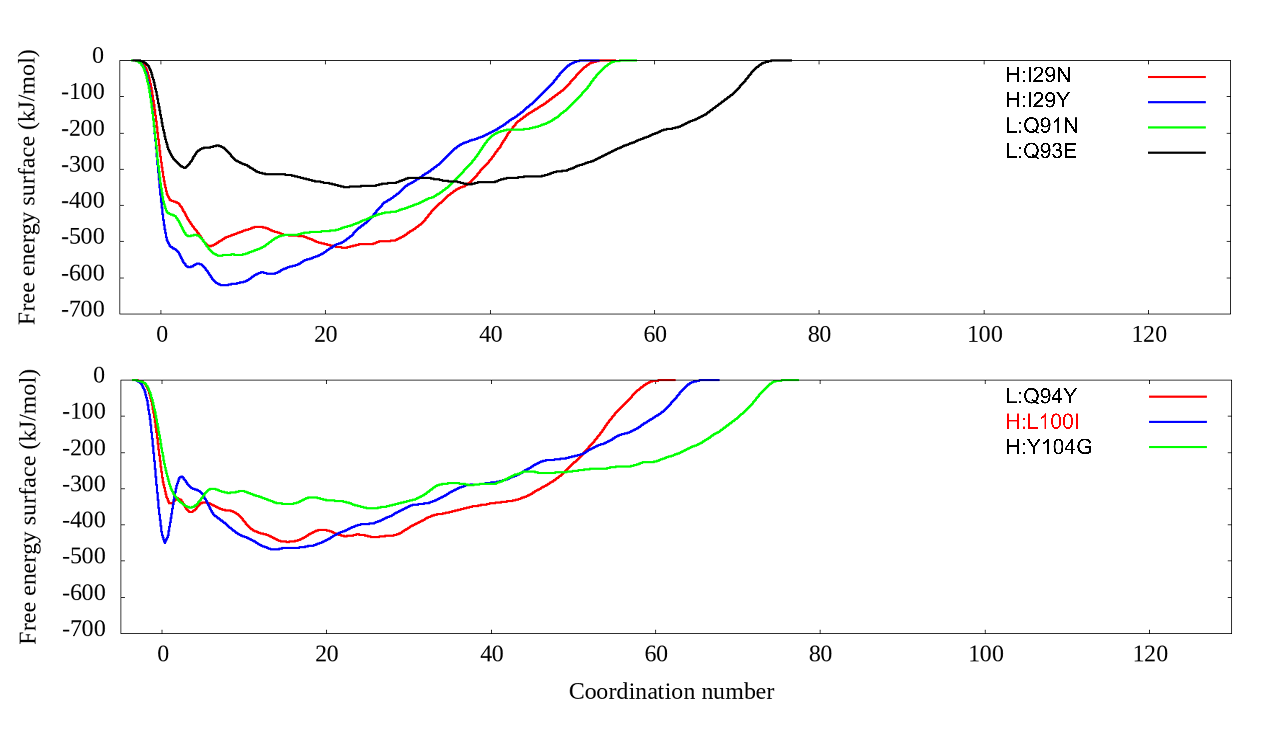
**

**Fig. S7. The free energy profiles caculated by metadynamics for the mutated Dr5 antibodies.**

**
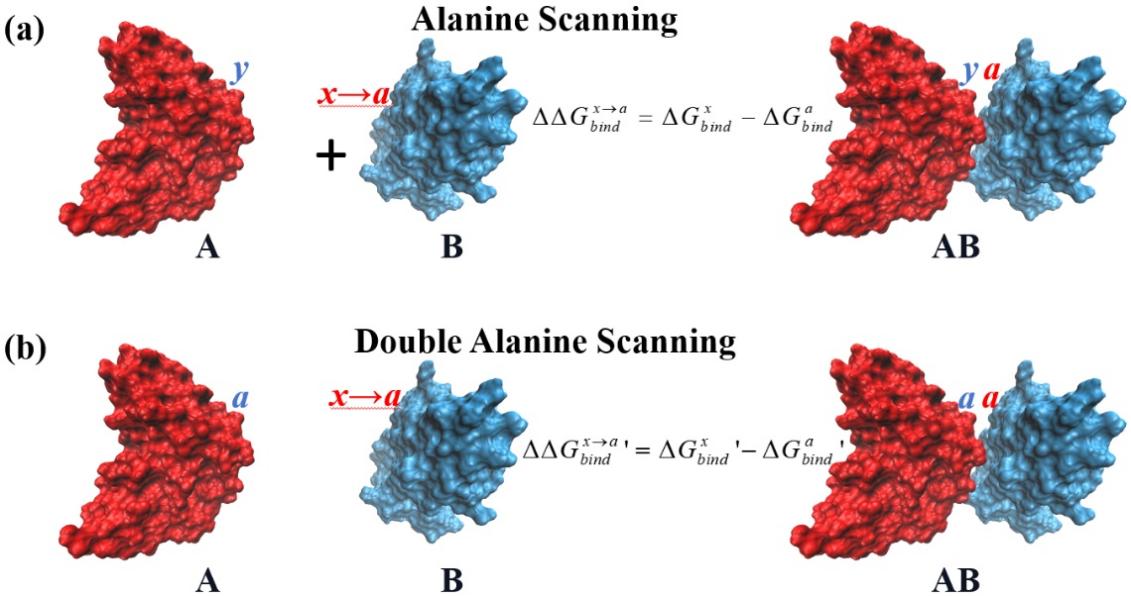
**

**Fig. S8. Alanine Scanning and Double Alanine Scanning method.**

**
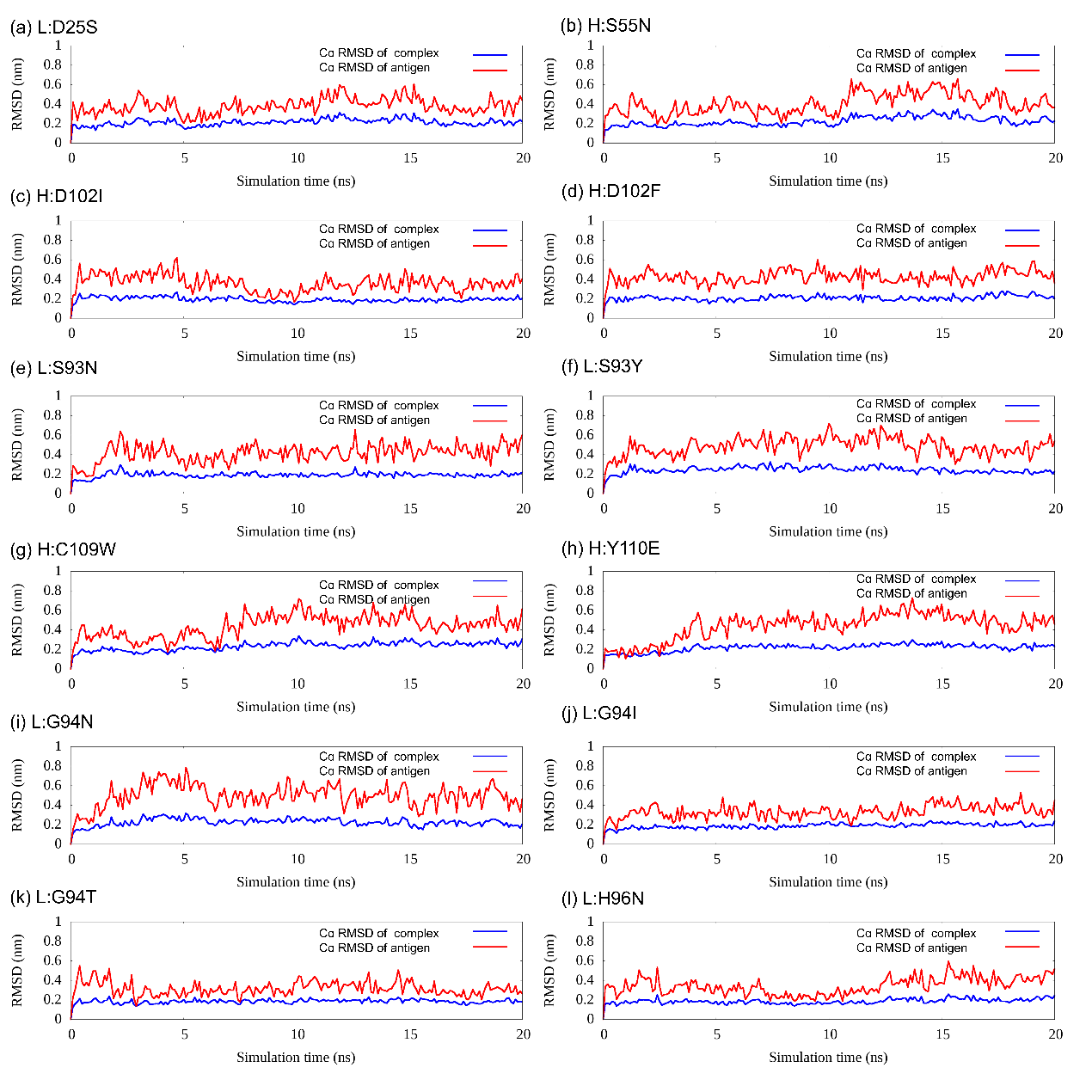
**

**Fig. S9. The RMSD value of antigen by fitting antibody and RMSD of complex during 60 ns MD simulation for the 3L11 designs.**


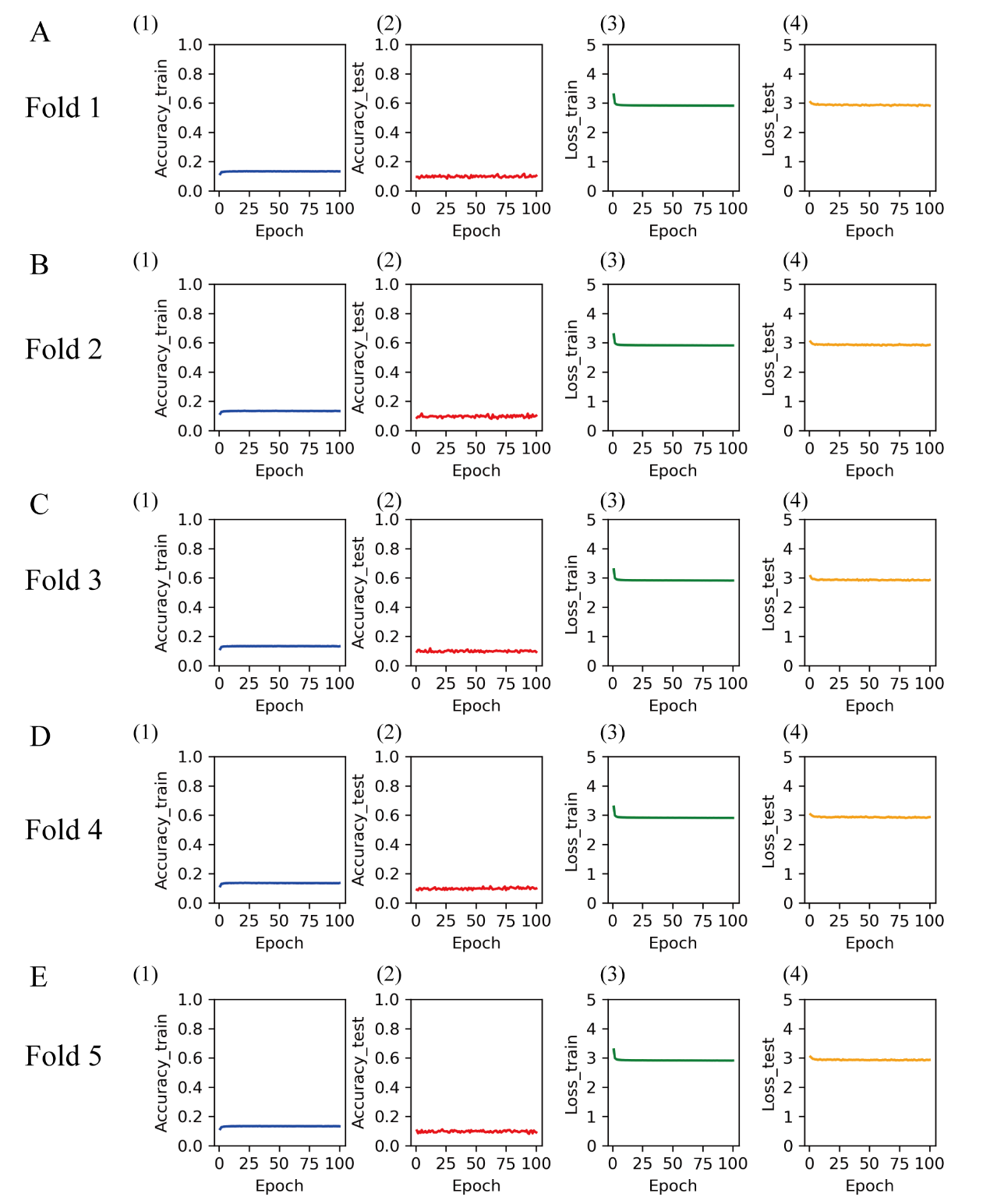


**Figure S10. Performance evaluation of MicrMutate through five-fold cross-validation.​​** The figure systematically presents the training dynamics across five independent folds (designated as ​​A. Fold 1​​, ​​B. Fold 2​​, ​​C. Fold 3​​, ​​D. Fold 4​​, and ​​E. Fold 5​​). Each fold comprises four unified subplots: (1) Training accuracy​​ (blue curves), (2) Test accuracy​​ (red curves), (3) Training loss​​ (green curves), and (4) Test loss​​ (orange curves), with all metrics tracked over 100 training epochs (x-axis: 0-100). Consistent patterns emerge across folds: training accuracy and loss exhibit rapid convergence followed by stabilization, while test metrics show marginally lower but stable performance.


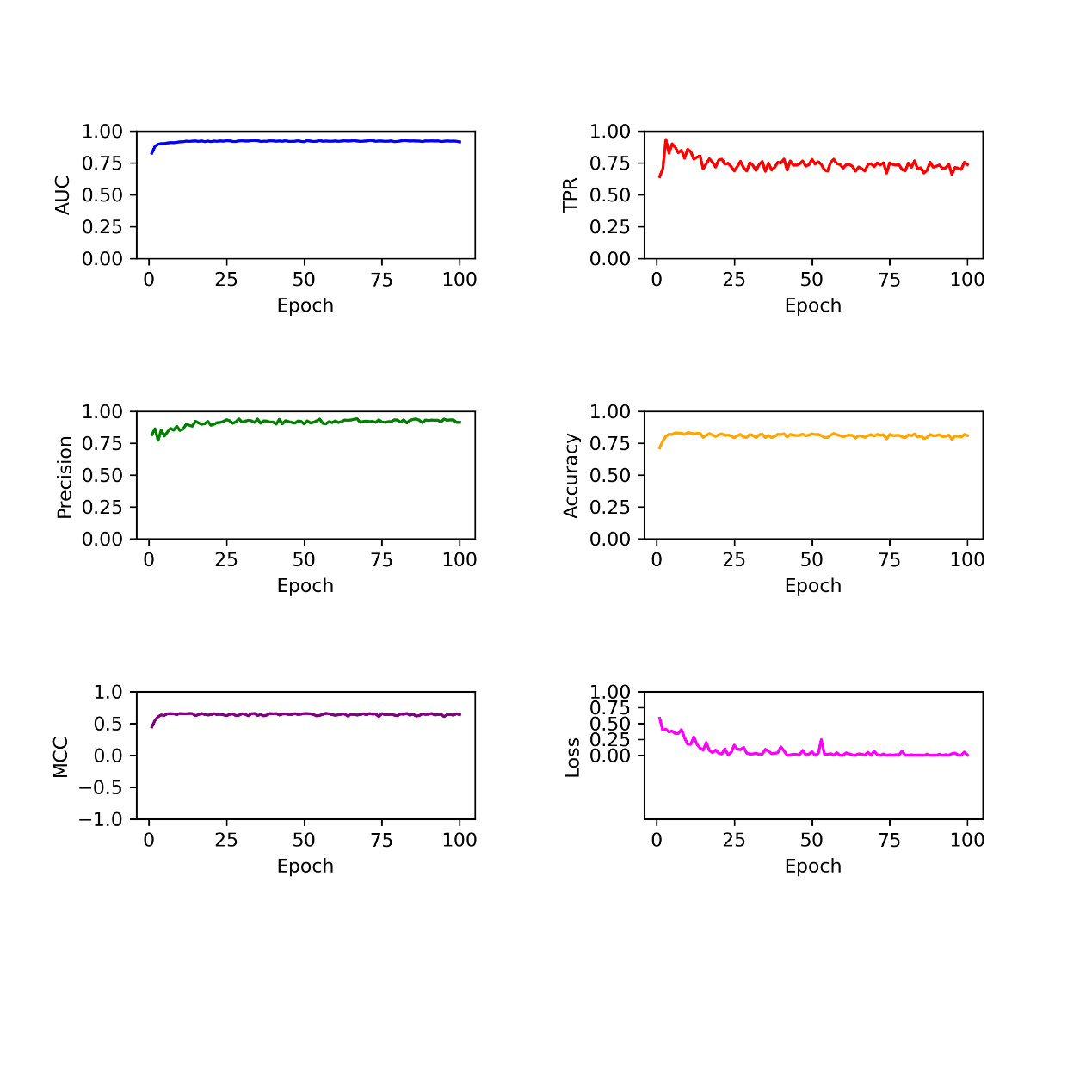


**Figure S11. the performance of Sgraph_AB with interface cutoff 0.5 Å over the testing set at different epochs during the training.**


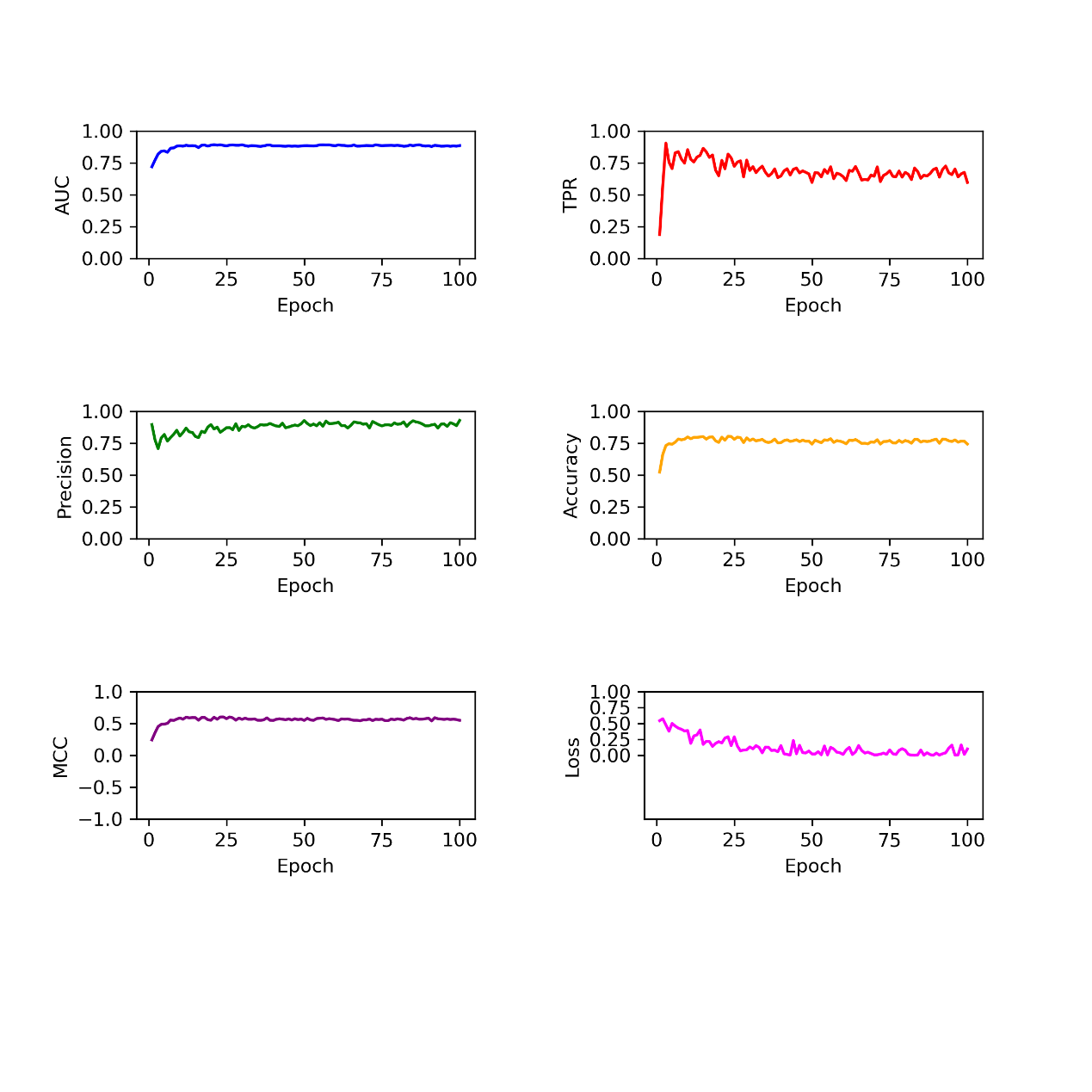


**Figure S12. the performance of Sgraph_AB with interface cutoff 1.2 Å over the testing set at different epochs during the training.**

**Supplementary Tables:**

**Table S1. The performance of Sgraph_AB compared with DeepGCN_Anti.**

| **Model (Epoch)** | **AUC** | **TPR** | **Precision** | **Accuracy** | **MCC** |
| --- | --- | --- | --- | --- | --- |
| Sgraph_AB(100) | 0.89 | 0.64 | 0.93 | 0.76 | 0.58 |
| DeepGCN_Anti(1000) | 0.83 | 0.49 | 0.89 | 0.68 | 0.45 |

**Table S2. The antibody sequences of 3L11 and mutated sequences.**

| **ID** | **Heavy Chain** | **Light Chain** |
| --- | --- | --- |
| 3L11 | QVQLVESGAEVKKPGASVKVSCKASGYIFTSYDINWVRQATGQGLEWMGWMNPDSGDTGFAQKFQGRVTMTRNTSITTAYMELSSLTSEDTAVYYCATGNADCSGGSCYNWFDPWGQGTLVTVSS | SSELTQDPAVSVALGQTVRITCQGDRLRSYYASWYQQKPGQAPVLVIYGKNNRPSGIPDRFSGSSSGNTASLTITGAQAEDEADYYCNSRDTSGYHLVFGGGTKLTVL |
| H:D102I | QVQLVESGAEVKKPGASVKVSCKASGYIFTSYDINWVRQATGQGLEWMGWMNPDSGDTGFAQKFQGRVTMTRNTSITTAYMELSSLTSEDTAVYYCATGNAICSGGSCYNWFDPWGQGTLVTVSS | SSELTQDPAVSVALGQTVRITCQGDRLRSYYASWYQQKPGQAPVLVIYGKNNRPSGIPDRFSGSSSGNTASLTITGAQAEDEADYYCNSRDTSGYHLVFGGGTKLTVL |
| H:D102F | QVQLVESGAEVKKPGASVKVSCKASGYIFTSYDINWVRQATGQGLEWMGWMNPDSGDTGFAQKFQGRVTMTRNTSITTAYMELSSLTSEDTAVYYCATGNAFCSGGSCYNWFDPWGQGTLVTVSS | SSELTQDPAVSVALGQTVRITCQGDRLRSYYASWYQQKPGQAPVLVIYGKNNRPSGIPDRFSGSSSGNTASLTITGAQAEDEADYYCNSRDTSGYHLVFGGGTKLTVL |
| L:S93N | QVQLVESGAEVKKPGASVKVSCKASGYIFTSYDINWVRQATGQGLEWMGWMNPDSGDTGFAQKFQGRVTMTRNTSITTAYMELSSLTSEDTAVYYCATGNADCSGGSCYNWFDPWGQGTLVTVSS | SSELTQDPAVSVALGQTVRITCQGDRLRSYYASWYQQKPGQAPVLVIYGKNNRPSGIPDRFSGSSSGNTASLTITGAQAEDEADYYCNSRDTNGYHLVFGGGTKLTVL |
| L:S93Y | QVQLVESGAEVKKPGASVKVSCKASGYIFTSYDINWVRQATGQGLEWMGWMNPDSGDTGFAQKFQGRVTMTRNTSITTAYMELSSLTSEDTAVYYCATGNADCSGGSCYNWFDPWGQGTLVTVSS | SSELTQDPAVSVALGQTVRITCQGDRLRSYYASWYQQKPGQAPVLVIYGKNNRPSGIPDRFSGSSSGNTASLTITGAQAEDEADYYCNSRDTYGYHLVFGGGTKLTVL |
| H:C109W | QVQLVESGAEVKKPGASVKVSCKASGYIFTSYDINWVRQATGQGLEWMGWMNPDSGDTGFAQKFQGRVTMTRNTSITTAYMELSSLTSEDTAVYYCATGNADCSGGSCWNWFDPWGQGTLVTVSS | SSELTQDPAVSVALGQTVRITCQGDRLRSYYASWYQQKPGQAPVLVIYGKNNRPSGIPDRFSGSSSGNTASLTITGAQAEDEADYYCNSRDTSGYHLVFGGGTKLTVL |
| H:Y110E | QVQLVESGAEVKKPGASVKVSCKASGYIFTSYDINWVRQATGQGLEWMGWMNPDSGDTGFAQKFQGRVTMTRNTSITTAYMELSSLTSEDTAVYYCATGNADCSGGSCYEWFDPWGQGTLVTVSS | SSELTQDPAVSVALGQTVRITCQGDRLRSYYASWYQQKPGQAPVLVIYGKNNRPSGIPDRFSGSSSGNTASLTITGAQAEDEADYYCNSRDTSGYHLVFGGGTKLTVL |
| L:G94N | QVQLVESGAEVKKPGASVKVSCKASGYIFTSYDINWVRQATGQGLEWMGWMNPDSGDTGFAQKFQGRVTMTRNTSITTAYMELSSLTSEDTAVYYCATGNADCSGGSCYNWFDPWGQGTLVTVSS | SSELTQDPAVSVALGQTVRITCQGDRLRSYYASWYQQKPGQAPVLVIYGKNNRPSGIPDRFSGSSSGNTASLTITGAQAEDEADYYCNSRDTSNYHLVFGGGTKLTVL |
| L:G94I | QVQLVESGAEVKKPGASVKVSCKASGYIFTSYDINWVRQATGQGLEWMGWMNPDSGDTGFAQKFQGRVTMTRNTSITTAYMELSSLTSEDTAVYYCATGNADCSGGSCYNWFDPWGQGTLVTVSS | SSELTQDPAVSVALGQTVRITCQGDRLRSYYASWYQQKPGQAPVLVIYGKNNRPSGIPDRFSGSSSGNTASLTITGAQAEDEADYYCNSRDTSIYHLVFGGGTKLTVL |
| L:G94T | QVQLVESGAEVKKPGASVKVSCKASGYIFTSYDINWVRQATGQGLEWMGWMNPDSGDTGFAQKFQGRVTMTRNTSITTAYMELSSLTSEDTAVYYCATGNADCSGGSCYNWFDPWGQGTLVTVSS | SSELTQDPAVSVALGQTVRITCQGDRLRSYYASWYQQKPGQAPVLVIYGKNNRPSGIPDRFSGSSSGNTASLTITGAQAEDEADYYCNSRDTSTYHLVFGGGTKLTVL |
| L:H96N | QVQLVESGAEVKKPGASVKVSCKASGYIFTSYDINWVRQATGQGLEWMGWMNPDSGDTGFAQKFQGRVTMTRNTSITTAYMELSSLTSEDTAVYYCATGNADCSGGSCYNWFDPWGQGTLVTVSS | SSELTQDPAVSVALGQTVRITCQGDRLRSYYASWYQQKPGQAPVLVIYGKNNRPSGIPDRFSGSSSGNTASLTITGAQAEDEADYYCNSRDTSGYNLVFGGGTKLTVL |
| L:D25S | QVQLVESGAEVKKPGASVKVSCKASGYIFTSYDINWVRQATGQGLEWMGWMNPDSGDTGFAQKFQGRVTMTRNTSITTAYMELSSLTSEDTAVYYCATGNADCSGGSCYNWFDPWGQGTLVTVSS | SSELTQDPAVSVALGQTVRITCQGSRLRSYYASWYQQKPGQAPVLVIYGKNNRPSGIPDRFSGSSSGNTASLTITGAQAEDEADYYCNSRDTSGYHLVFGGGTKLTVL |
| H:S55N | QVQLVESGAEVKKPGASVKVSCKASGYIFTSYDINWVRQATGQGLEWMGWMNPDNGDTGFAQKFQGRVTMTRNTSITTAYMELSSLTSEDTAVYYCATGNADCSGGSCYNWFDPWGQGTLVTVSS | SSELTQDPAVSVALGQTVRITCQGDRLRSYYASWYQQKPGQAPVLVIYGKNNRPSGIPDRFSGSSSGNTASLTITGAQAEDEADYYCNSRDTSGYHLVFGGGTKLTVL |

**Table S3. The antibody sequences of Dr5 antibody (IgG1-hDR5-01) and mutated sequences.**

| **ID** | **Heavy Chain** | **Light Chain** |
| --- | --- | --- |
| IgG1-hDR5-01 | EVQLQQSGAEVVKPGASVKLSCKASGFNIKDTFIHWVKQAPGQGLEWIGRIDPANTNTKYDPKFQGKATITTDTSSNTAYMELSSLRSEDTAVYYCVRGLYTYYFDYWGQGTLVTVSS | EIVMTQSPATLSVSPGERATLSCRASQSISNNLHWYQQKPGQAPRLLIKFASQSITGIPARFSGSGSGTEFTLTISSLQSEDFAVYYCQQGNSWPYTFGQGTKLEIKRTV |
| H:L100I | EVQLQQSGAEVVKPGASVKLSCKASGFNIKDTFIHWVKQAPGQGLEWIGRIDPANTNTKYDPKFQGKATITTDTSSNTAYMELSSLRSEDTAVYYCVRGIYTYYFDYWGQGTLVTVSS | EIVMTQSPATLSVSPGERATLSCRASQSISNNLHWYQQKPGQAPRLLIKFASQSITGIPARFSGSGSGTEFTLTISSLQSEDFAVYYCQQGNSWPYTFGQGTKLEIKRTV |
| H:Y104G | EVQLQQSGAEVVKPGASVKLSCKASGFNIKDTFIHWVKQAPGQGLEWIGRIDPANTNTKYDPKFQGKATITTDTSSNTAYMELSSLRSEDTAVYYCVRGLYTYGFDYWGQGTLVTVSS | EIVMTQSPATLSVSPGERATLSCRASQSISNNLHWYQQKPGQAPRLLIKFASQSITGIPARFSGSGSGTEFTLTISSLQSEDFAVYYCQQGNSWPYTFGQGTKLEIKRTV |
| H:I29N | EVQLQQSGAEVVKPGASVKLSCKASGFNNKDTFIHWVKQAPGQGLEWIGRIDPANTNTKYDPKFQGKATITTDTSSNTAYMELSSLRSEDTAVYYCVRGLYTYYFDYWGQGTLVTVSS | EIVMTQSPATLSVSPGERATLSCRASQSISNNLHWYQQKPGQAPRLLIKFASQSITGIPARFSGSGSGTEFTLTISSLQSEDFAVYYCQQGNSWPYTFGQGTKLEIKRTV |
| H:I29Y | EVQLQQSGAEVVKPGASVKLSCKASGFNYKDTFIHWVKQAPGQGLEWIGRIDPANTNTKYDPKFQGKATITTDTSSNTAYMELSSLRSEDTAVYYCVRGLYTYYFDYWGQGTLVTVSS | EIVMTQSPATLSVSPGERATLSCRASQSISNNLHWYQQKPGQAPRLLIKFASQSITGIPARFSGSGSGTEFTLTISSLQSEDFAVYYCQQGNSWPYTFGQGTKLEIKRTV |
| L:Q91N | EVQLQQSGAEVVKPGASVKLSCKASGFNIKDTFIHWVKQAPGQGLEWIGRIDPANTNTKYDPKFQGKATITTDTSSNTAYMELSSLRSEDTAVYYCVRGLYTYYFDYWGQGTLVTVSS | EIVMTQSPATLSVSPGERATLSCRASQSISNNLHWYQQKPGQAPRLLIKFASQSITGIPARFSGSGSGTEFTLTISSLQSEDFAVYYCQQNNSWPYTFGQGTKLEIKRTV |
| L:Q93E | EVQLQQSGAEVVKPGASVKLSCKASGFNIKDTFIHWVKQAPGQGLEWIGRIDPANTNTKYDPKFQGKATITTDTSSNTAYMELSSLRSEDTAVYYCVRGLYTYYFDYWGQGTLVTVSS | EIVMTQSPATLSVSPGERATLSCRASQSISNNLHWYQQKPGQAPRLLIKFASQSITGIPARFSGSGSGTEFTLTISSLQSEDFAVYYCQQGNEWPYTFGQGTKLEIKRTV |
| L:Q94Y | EVQLQQSGAEVVKPGASVKLSCKASGFNIKDTFIHWVKQAPGQGLEWIGRIDPANTNTKYDPKFQGKATITTDTSSNTAYMELSSLRSEDTAVYYCVRGLYTYYFDYWGQGTLVTVSS | EIVMTQSPATLSVSPGERATLSCRASQSISNNLHWYQQKPGQAPRLLIKFASQSITGIPARFSGSGSGTEFTLTISSLQSEDFAVYYCQQGNSYPYTFGQGTKLEIKRTV |

**Table S4. The interaction free energy dG between antibody and antigen and all detailed energy terms (kcal/mol).**

| WT | | | H :55ASN | | | H:55THR | | | L:93TYR | | | L:94ASN | | |
| --- | --- | --- | --- | --- | --- | --- | --- | --- | --- | --- | --- | --- | --- | --- |
| Mut-Wid | dH | dIE | Mut-Wid | dH | dIE | Mut-Wid | dH | dIE | Mut-Wid | dH | dIE | Mut-Wid | dH | dIE |
| H:32TYR | -0.64 | 0.23 | H:32TYR | -0.35 | 0.26 | H:32TYR | -5.31 | 1.47 | H:32TYR | -0.18 | 0.17 | H:32TYR | -0.18 | 0.02 |
| H:33ASP | -2.83 | 2.31 | H:33ASP | -2.20 | 3.34 | H:33ASP | -2.16 | 3.07 | H:33ASP | -1.67 | 1.16 | H:33ASP | -2.26 | 3.08 |
| H:35ASN | -0.33 | 0.20 | H:35ASN | -1.11 | 0.36 | H:35ASN | -1.09 | 0.66 | H:35ASN | -0.02 | 0.02 | H:35ASN | -1.77 | 0.55 |
| H:47TRP | -1.51 | 0.65 | H:47TRP | -1.20 | 0.25 | H:47TRP | -0.56 | 0.13 | H:47TRP | -0.10 | 0.04 | H:47TRP | -0.86 | 0.14 |
| H:50TRP | -9.44 | 1.63 | H:50TRP | -5.92 | 1.01 | H:50TRP | -6.62 | 1.27 | H:50TRP | -3.11 | 1.51 | H:50TRP | -6.64 | 1.45 |
| H:52ASN | -1.14 | 0.22 | H:52ASN | -0.99 | 1.13 | H:52ASN | -0.35 | 0.36 | H:52ASN | -0.43 | 0.88 | H:52ASN | -1.04 | 0.28 |
| H:55SER | -- | -- | H:55ASN | -0.25 | 0.42 | H:55THR | -0.13 | 0.07 | H:55SER | -- | -- | H:55SER | -- | -- |
| H:57ASP | -3.46 | 3.20 | H:57ASP | -1.30 | 0.38 | H:57ASP | -1.43 | 0.36 | H:57ASP | -0.64 | 1.61 | H:57ASP | -1.32 | 0.25 |
| H:58THR | -0.15 | 0.04 | H:58THR | -0.03 | 0.00 | H:58THR | -0.04 | 0.01 | H:58THR | -0.02 | 0.00 | H:58THR | -0.04 | 0.01 |
| H:102ASP | -2.74 | 2.66 | H:102ASP | -1.14 | 0.63 | H:102ASP | -1.04 | 0.20 | H:102ASP | -1.69 | 2.25 | H:102ASP | -0.50 | 0.30 |
| H:103CYX | -0.02 | 0.05 | H:103CYX | -0.06 | 0.10 | H:103CYX | -0.14 | 0.20 | H:103CYX | 0.12 | -0.02 | H:103CYX | -1.18 | 0.67 |
| H:104SER | -2.48 | 2.56 | H:104SER | 0.02 | 0.04 | H:104SER | -0.84 | 0.10 | H:104SER | -1.46 | 0.21 | H:104SER | -1.83 | 1.99 |
| H:107SER | 0.01 | 0.11 | H:107SER | -0.38 | 0.95 | H:107SER | -1.94 | 0.55 | H:107SER | 0.00 | 0.06 | H:107SER | -0.90 | 0.41 |
| H:108CYX | 0.07 | 0.03 | H:108CYX | -0.03 | 0.05 | H:108CYX | 0.06 | 0.02 | H:108CYX | 0.13 | 0.12 | H:108CYX | -2.49 | 0.17 |
| H:109TYR | -5.10 | 0.70 | H:109TYR | -6.18 | 2.82 | H:109TYR | -0.35 | 0.11 | H:109TYR | -7.42 | 0.83 | H:109TYR | -3.23 | 0.37 |
| L:26ARG | -0.17 | 0.40 | L:26ARG | 0.42 | 0.04 | L:26ARG | -0.03 | 0.51 | L:26ARG | 0.25 | 0.22 | L:26ARG | 0.39 | 0.06 |
| L:28ARG | 0.45 | 0.14 | L:28ARG | -0.10 | 0.55 | L:28ARG | 0.06 | 0.58 | L:28ARG | -3.97 | 4.20 | L:28ARG | -0.68 | 0.72 |
| L:29SER | -0.03 | 0.06 | L:29SER | -0.46 | 0.10 | L:29SER | -0.29 | 0.49 | L:29SER | -0.56 | 1.88 | L:29SER | -0.21 | 0.25 |
| L:30TYR | -0.68 | 0.09 | L:30TYR | -3.13 | 0.61 | L:30TYR | -4.10 | 0.57 | L:30TYR | -0.98 | 0.14 | L:30TYR | -5.03 | 1.57 |
| L:31TYR | -0.82 | 0.25 | L:31TYR | -0.59 | 0.08 | L:31TYR | -0.14 | 0.09 | L:31TYR | -2.54 | 1.67 | L:31TYR | -3.98 | 0.90 |
| L:50LYS | 0.42 | 0.13 | L:50LYS | 0.06 | 0.34 | L:50LYS | 0.42 | 0.07 | L:50LYS | 0.00 | 0.34 | L:50LYS | 0.26 | 0.38 |
| L:51ASN | 0.01 | 0.01 | L:51ASN | 0.04 | 0.12 | L:51ASN | 0.01 | 0.01 | L:51ASN | 0.01 | 0.22 | L:51ASN | 0.02 | 0.04 |
| L:52ASN | 0.03 | 0.03 | L:52ASN | 0.06 | 0.14 | L:52ASN | 0.02 | 0.02 | L:52ASN | 0.04 | 0.20 | L:52ASN | 0.03 | 0.05 |
| L:90ARG | -3.41 | 0.82 | L:90ARG | -6.24 | 2.43 | L:90ARG | -4.44 | 2.51 | L:90ARG | 0.27 | 0.12 | L:90ARG | -3.85 | 2.53 |
| L:92THR | -0.28 | 0.17 | L:92THR | -0.02 | 0.02 | L:92THR | -0.46 | 0.31 | L:92THR | -0.05 | 0.02 | L:92THR | -0.67 | 0.43 |
| L:93SER | -0.08 | 0.03 | L:93SER | 0.02 | 0.04 | L:93SER | -0.27 | 0.42 | L:93TYR | -3.98 | 0.68 | L:93SER | -0.07 | 0.01 |
| L:94GLY | -- | -- | L:94GLY | -- | -- | L:94GLY | -- | -- | L:94GLY | -- | -- | L:94ASN | -3.76 | 0.95 |
| L:95TYR | -2.44 | 1.07 | L:95TYR | -5.31 | 0.93 | L:95TYR | -9.76 | 2.06 | L:95TYR | -5.61 | 2.11 | L:95TYR | -4.34 | 3.04 |
| L:96HIE | -0.22 | 0.10 | L:96HIE | -0.13 | 0.12 | L:96HIE | -0.31 | 0.21 | L:96HIE | -0.03 | 0.02 | L:96HIE | -0.26 | 0.10 |
| A:82PRO | -0.07 | 0.07 | A:82PRO | -0.43 | 0.38 | A:82PRO | -1.64 | 0.32 | A:82PRO | -0.14 | 0.09 | A:82PRO | -0.59 | 0.12 |
| A:84CYX | -- | -- | A:84CYX | -- | -- | A:84CYX | -- | -- | A:84CYX | 0.03 | 0.00 | A:84CYX | 0.02 | 0.00 |
| A:85ASP | -0.71 | 0.47 | A:85ASP | -1.33 | 1.63 | A:85ASP | -0.29 | 2.19 | A:85ASP | -0.12 | 1.72 | A:85ASP | -1.20 | 2.41 |
| A:86GLN | -0.91 | 1.58 | A:86GLN | -1.88 | 1.36 | A:86GLN | -3.77 | 3.11 | A:86GLN | -1.75 | 0.40 | A:86GLN | -5.16 | 1.40 |
| A:87PHE | 0.01 | 0.00 | A:87PHE | -0.09 | 0.04 | A:87PHE | -0.35 | 0.07 | A:87PHE | -0.18 | 0.00 | A:87PHE | -1.09 | 0.27 |
| A:88LEU | -0.13 | 0.02 | A:88LEU | -0.19 | 0.04 | A:88LEU | -0.17 | 0.00 | A:88LEU | -0.51 | 0.03 | A:88LEU | -0.64 | 0.05 |
| A:89GLU | -0.31 | 0.27 | A:89GLU | -0.40 | 0.46 | A:89GLU | -0.05 | 0.17 | A:89GLU | -1.99 | 2.12 | A:89GLU | 0.14 | 0.36 |
| A:127ASP | 0.05 | 0.08 | A:127ASP | -0.01 | 0.09 | A:127ASP | 0.10 | 0.05 | A:127ASP | -0.52 | 1.87 | A:127ASP | 0.23 | 0.06 |
| A:128LYS | -0.24 | 0.08 | A:128LYS | -0.10 | 0.09 | A:128LYS | -0.23 | 0.06 | A:128LYS | -0.16 | 0.10 | A:128LYS | -0.32 | 0.10 |
| A:129GLU | 0.05 | 0.12 | A:129GLU | 0.07 | 0.09 | A:129GLU | 0.17 | 0.04 | A:129GLU | -1.26 | 1.89 | A:129GLU | 0.30 | 0.05 |
| A:133PHE | -0.25 | 0.08 | A:133PHE | 0.00 | 0.01 | A:133PHE | 0.00 | 0.00 | A:133PHE | 0.00 | 0.04 | A:133PHE | -0.02 | 0.01 |
| A:134THR | -0.11 | 0.37 | A:134THR | 0.02 | 0.01 | A:134THR | 0.00 | 0.01 | A:134THR | 0.02 | 0.05 | A:134THR | 0.00 | 0.01 |
| A:135TYR | -0.36 | 0.08 | A:135TYR | -0.08 | 0.00 | A:135TYR | -0.02 | 0.00 | A:135TYR | -0.10 | 0.01 | A:135TYR | -0.02 | 0.00 |
| A:139ARG | -4.26 | 2.66 | A:139ARG | -1.10 | 0.74 | A:139ARG | -0.74 | 0.14 | A:139ARG | -1.34 | 1.34 | A:139ARG | -0.72 | 0.19 |
| A:140THR | -0.55 | 1.14 | A:140THR | -0.51 | 0.58 | A:140THR | 0.01 | 0.04 | A:140THR | -0.41 | 0.52 | A:140THR | -0.04 | 0.07 |
| A:141ASN | -2.44 | 1.72 | A:141ASN | -2.89 | 1.32 | A:141ASN | -3.02 | 2.09 | A:141ASN | -3.18 | 1.27 | A:141ASN | 0.02 | 0.26 |
| A:144THR | -0.09 | 0.06 | A:144THR | -0.06 | 0.08 | A:144THR | -0.10 | 0.05 | A:144THR | -0.02 | 0.01 | A:144THR | -0.04 | 0.05 |
| A:149ARG | -6.35 | 2.91 | A:149ARG | -9.88 | 2.82 | A:149ARG | -8.49 | 2.69 | A:149ARG | -2.70 | 3.09 | A:149ARG | -10.98 | 2.81 |
| A:149ARG | -2.76 | 0.68 | A:149ARG | -2.59 | 0.28 | A:149ARG | -6.78 | 0.70 | A:149ARG | -4.51 | 0.38 | A:149ARG | -8.47 | 0.67 |
| A:150SER | -1.48 | 0.44 | A:150SER | -2.34 | 0.77 | A:150SER | -1.21 | 0.34 | A:150SER | -1.15 | 0.78 | A:150SER | -1.88 | 0.17 |
| A:152SER | -1.75 | 0.93 | A:152SER | -1.57 | 1.07 | A:152SER | -0.69 | 1.40 | A:152SER | -0.04 | 0.32 | A:152SER | -1.97 | 0.92 |
| A:153SER | -0.31 | 0.01 | A:153SER | -0.18 | 0.10 | A:153SER | -0.54 | 0.15 | A:153SER | -0.64 | 0.04 | A:153SER | -1.38 | -0.16 |
| A:154PHE | -0.14 | 0.02 | A:154PHE | -0.21 | 0.01 | A:154PHE | -0.81 | 0.23 | A:154PHE | -0.36 | 0.02 | A:154PHE | -0.75 | 0.04 |
| A:155TYR | -0.08 | 0.00 | A:155TYR | -0.09 | 0.01 | A:155TYR | -0.22 | 0.01 | A:155TYR | -0.12 | 0.00 | A:155TYR | -0.21 | 0.02 |
| A:157GLU | -0.13 | 0.18 | A:157GLU | -0.04 | 0.14 | A:157GLU | 0.15 | 0.08 | A:157GLU | -0.55 | 0.19 | A:157GLU | -0.24 | 0.20 |
| A:158MET | 0.01 | 0.04 | A:158MET | 0.01 | 0.03 | A:158MET | -0.02 | 0.04 | A:158MET | 0.00 | 0.03 | A:158MET | -0.11 | 0.01 |
| A:159LYS | -2.93 | 2.66 | A:159LYS | -1.74 | 0.73 | A:159LYS | -2.03 | 0.73 | A:159LYS | -3.62 | 1.50 | A:159LYS | -1.56 | 0.67 |
| A:160TRP | -0.04 | 0.10 | A:160TRP | -0.01 | 0.06 | A:160TRP | -0.05 | 0.03 | A:160TRP | -0.05 | 0.03 | A:160TRP | -0.04 | 0.04 |
| A:162LEU | -0.07 | 0.02 | A:162LEU | -0.03 | 0.01 | A:162LEU | -0.01 | 0.01 | A:162LEU | -0.02 | 0.00 | A:162LEU | 0.03 | 0.01 |
| A:264ASP | -0.88 | 2.49 | A:264ASP | 0.05 | 0.19 | A:264ASP | 0.30 | 0.09 | A:264ASP | -0.60 | 2.02 | A:264ASP | 0.02 | 0.85 |
| A:265ARG | -2.52 | 0.48 | A:265ARG | -0.21 | 0.29 | A:265ARG | -0.35 | 0.09 | A:265ARG | -3.83 | 0.63 | A:265ARG | -0.99 | 0.33 |
| A:270ARG | -0.08 | 0.08 | A:270ARG | -0.01 | 0.12 | A:270ARG | -0.07 | 0.08 | A:270ARG | -0.10 | 0.19 | A:270ARG | -0.18 | 0.07 |

**Table S5. To compare with the wild-type, the top 10 residue pairs with enhanced interactions in the mutated form and their relative changes in distance.**

| Mutation | R1-Num | R2-Num | R1-Type | R2-Type | dDist | ddH |
| --- | --- | --- | --- | --- | --- | --- |
| H:S55N | H:50 | A:148 | TRP | ARG | -0.71 | -2.33 |
|  | L:30 | A:86 | TYR | GLN | -5.15 | -0.86 |
|  | L:28 | A:86 | ARG | GLN | -5.92 | -0.80 |
|  | L:95 | A:148 | TYR | ARG | -1.41 | -0.69 |
|  | H:109 | A:141 | TYR | ASN | -3.14 | -0.64 |
|  | L:29 | A:86 | SER | GLN | -4.18 | -0.55 |
|  | H:33 | A:148 | ASP | ARG | -5.4 | -0.47 |
|  | L:90 | A:149 | ARG | ARG | -0.39 | -0.44 |
|  | H:57 | A:148 | ASP | ARG | 1.95 | -0.41 |
|  | H:104 | A:264 | SER | ASP | 13.08 | -0.41 |
| H:S55T | H:50 | A:148 | TRP | ARG | -1.94 | -3.14 |
|  | L:30 | A:86 | TYR | GLN | -5.13 | -0.86 |
|  | L:90 | A:149 | ARG | ARG | -0.4 | -0.64 |
|  | H:107 | A:149 | SER | ARG | -4.32 | -0.57 |
|  | H:104 | A:264 | SER | ASP | 8.92 | -0.41 |
|  | L:26 | A:86 | ARG | GLN | -1.11 | -0.32 |
|  | L:90 | A:82 | ARG | PRO | -2.35 | -0.29 |
|  | H:102 | A:264 | ASP | ASP | 8.29 | -0.26 |
|  | L:30 | A:85 | TYR | ASP | -1.41 | -0.21 |
|  | H:32 | A:150 | TYR | SER | -9.06 | -0.21 |
| L:S93Y | L:93 | A:86 | TYR | GLN | -3.56 | -1.28 |
|  | L:26 | A:86 | ARG | GLN | -1.89 | -0.97 |
|  | L:28 | A:89 | ARG | GLU | -7.96 | -0.93 |
|  | H:109 | A:149 | TYR | ARG | -4.18 | -0.87 |
|  | L:30 | A:149 | TYR | ARG | -2.37 | -0.64 |
|  | H:109 | A:153 | TYR | SER | -3.47 | -0.64 |
|  | H:107 | A:149 | SER | ARG | -4.23 | -0.56 |
|  | H:57 | A:148 | ASP | ARG | 2.09 | -0.50 |
|  | L:31 | A:159 | TYR | LYS | -6.48 | -0.48 |
|  | L:50 | A:264 | LYS | ASP | -9.3 | -0.42 |
| L:G94N | H:50 | A:148 | TRP | ARG | -2.01 | -2.93 |
|  | L:95 | A:148 | TYR | ARG | -3.41 | -0.91 |
|  | L:31 | A:86 | TYR | GLN | -9.69 | -0.87 |
|  | H:108 | A:149 | CYX | ARG | -10.11 | -0.79 |
|  | H:104 | A:264 | SER | ASP | -0.93 | -0.56 |
|  | H:33 | A:152 | ASP | SER | -0.13 | -0.49 |
|  | L:90 | A:149 | ARG | ARG | 0.22 | -0.40 |
|  | H:109 | A:150 | TYR | SER | -3.59 | -0.40 |
|  | H:108 | A:85 | CYX | ASP | -11.48 | -0.35 |
|  | H:35 | A:148 | ASN | ARG | -4.96 | -0.33 |

**Table S6. Average Cα RMSD (Å) of the antigen (alignment reference: antibody Cα) during the 60 ns MD simulation. The standard deviation of RMSD (STD RMSD) are also given.**

| **Mutated Antibody** | **Mean RMSD (nm)** | **STD RMSD (nm)** |
| --- | --- | --- |
| **H:S55N** | **0.34** | **0.11** |
| **L:G94N** | **0.36** | **0.08** |
| **L:S93Y** | **0.40** | **0.09** |
| H:C109W | 0.43 | 0.09 |
| H:D102F | 0.52 | 0.13 |
| L:G94I | 0.53 | 0.21 |
| H:Y110E | 0.56 | 0.12 |
| L:D25S | 0.600029 | 0.176881 |
| H:D102I | 0.691982 | 0.172775 |
| **L:S93N** | **0.770509** | **0.427582** |
| L:H96N | 0.780644 | 0.29929 |
| L:G94T | 1.005991 | 0.211883 |

**Table S7: Five-Fold Cross-validation Results for MicrMutate (Accuracy and Loss).**

| **Fold** | **Epoch** | **Train Accuracy** | **Test Accuracy** | **Train loss** | **Test loss** |
| --- | --- | --- | --- | --- | --- |
| Fold_1 | 100 | 0.133 | 0.104 | 2.909 | 2.910 |
| Fold_2 | 100 | 0.134 | 0.103 | 2.909 | 2.930 |
| Fold_3 | 100 | 0.133 | 0.095 | 2.910 | 2.929 |
| Fold_4 | 100 | 0.137 | 0.100 | 2.907 | 2.935 |
| Fold_5 | 100 | 0.133 | 0.092 | 2.911 | 2.934 |
| Average | 100 | 0.134 | 0.099 | 2.909 | 2.928 |

**Table S8. The performance metrics of Sgraph_Anti when using interface cutoff of 0.5 Å, 0.9 Å and 1.2 Å as interface cutoff.**

| **Interface cutoff（Å）** | **Epoch** | **AUC** | **TPR** | **Precision** | **Accuracy** | **MCC** | **Loss** |
| --- | --- | --- | --- | --- | --- | --- | --- |
| 0.5 | 1 | 0.827 | 0.642 | 0.817 | 0.713 | 0.448 | 0.582 |
|  | 20 | 0.917 | 0.772 | 0.890 | 0.815 | 0.638 | 0.035 |
|  | 40 | 0.923 | 0.750 | 0.916 | 0.817 | 0.652 | 0.132 |
|  | 60 | 0.921 | 0.708 | 0.925 | 0.800 | 0.630 | 0.004 |
|  | 80 | 0.918 | 0.688 | 0.931 | 0.793 | 0.621 | 0.001 |
|  | 100 | 0.915 | 0.736 | 0.914 | 0.809 | 0.638 | 0.005 |
|  | 1 | 0.828 | 0.816 | 0.765 | 0.752 | 0.488 | 0.458 |
| 0.9 | 20 | 0.893 | 0.675 | 0.898 | 0.770 | 0.572 | 0.225 |
|  | 40 | 0.891 | 0.676 | 0.900 | 0.772 | 0.575 | 0.102 |
|  | 60 | 0.890 | 0.698 | 0.898 | 0.782 | 0.590 | 0.035 |
|  | 80 | 0.889 | 0.589 | 0.941 | 0.744 | 0.557 | 0.005 |
|  | 100 | 0.890 | 0.636 | 0.927 | 0.763 | 0.576 | 0.003 |
| 1.2 | 1 | 0.720 | 0.188 | 0.896 | 0.523 | 0.242 | 0.544 |
|  | 20 | 0.889 | 0.649 | 0.896 | 0.756 | 0.551 | 0.214 |
|  | 40 | 0.883 | 0.647 | 0.894 | 0.754 | 0.546 | 0.152 |
|  | 60 | 0.885 | 0.642 | 0.907 | 0.758 | 0.559 | 0.013 |
|  | 80 | 0.888 | 0.676 | 0.898 | 0.771 | 0.573 | 0.079 |
|  | 100 | 0.886 | 0.597 | 0.929 | 0.743 | 0.549 | 0.099 |
